# Modeling Peptides in Aqueous Ionic Liquid Mixtures: A Quantum Mechanics/Molecular Dynamics Study of Structural and NMR Properties

**DOI:** 10.1021/acs.jctc.6c01192

**Published:** 2026-08-01

**Authors:** Žyginta Murnikova, Kęstutis Aidas

**Affiliations:** Institute of Chemical Physics, Faculty of Physics, Vilnius University, Saulėtekio ave. 3, Vilnius 10257, Lithuania

## Abstract

In this pilot study, structural properties and ^1^H NMR chemical shifts of an Ala-Glu-Pro-Phe peptide dissolved in an aqueous solution and an aqueous mixture of the 1-ethyl-3-methylimidazolium trifluoroacetate ionic liquid (IL) have been scrutinized using an integrated computational protocol based on classical molecular dynamics (MD) simulations and combined quantum mechanics/molecular mechanics (QM/MM) approaches for NMR shielding constants. Two long-lived trans and cis Pro isomers of the tetrapeptide have been considered. MD simulations as long as 400 ns were found to be too short to ensure complete sampling of the conformational phase space of the peptide in aqueous IL solution, and thus, the analysis was performed for four distinct conformations of either isomer of the peptide separately. Solvent molecules within the first solvation shell of the solute were treated quantum mechanically in the QM/MM calculations of ^1^H NMR shieldings. The constituent ions of the IL were seen to condense around the tetrapeptide, abundantly displacing water molecules from its first solvation shell, and the preference for the imidazolium cations to condense around the peptide in solution was identified. Prominent hydrogen bonding together with π–π stacking interactions between the benzene ring in the Phe residue and the imidazolium ring of the cations assist in maintaining the ionic cage that surrounds the peptide. Accordingly, the computed ^1^H NMR signals of the peptide in aqueous IL solution are shifted w.r.t. their values in aqueous solution. The computational results allow identifying several ^1^H NMR signals which could be potential spectral NMR markers of the conformational or the isomeric state of the tetrapeptide.

## Graphical abstract

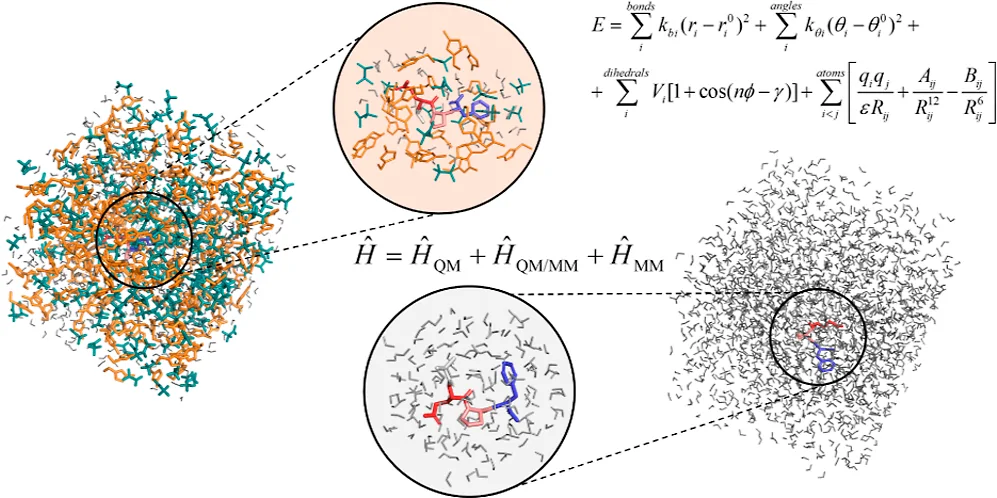

## 1 Introduction

One of the main advantages of room-temperature ionic liquids (ILs)—molten salts composed of asymmetric bulky cations and organic or inorganic anions^1,2^ —is their ability to dissolve a diverse range of chemical compounds.^3^ The solvating capability of ILs stems from the fact that various intermolecular interactions can be established between the solute and the constituent ions of an IL, including electrostatic and van der Waals type, as well as specific hydrogen bonding and π–π stacking interactions. On top of that, amphiphilicity is yet another property that especially the IL cations often possess, thus allowing both hydrophobic and hydrophilic molecules to dissolve in ILs, rendering them particularly attractive in biochemical and biomedical applications.^4–11^ ILs are also being actively assessed as a potential medium for long-term storage of biological macromolecules—proteins and nucleic acids.^12,13^ It has been demonstrated that some proteins such as cytochrome C, α-chymotrypsin, or monellin may retain their native structure and function in aqueous mixtures of ILs over significantly extended periods of time.^12^ Furthermore, IL solutions may not only stabilize enzymes but also lead to the enhancement of their catalytic activity, as exemplified by the case of lysozyme.^14^ ILs may also prevent protein aggregation and even trigger aggregation reversal and refolding.^6^ Suppression of insulin fibrillation in certain IL systems has been recently demonstrated,^15^ hoping that new ILs could potentially be developed and used to prevent fibril formation^16^ or to facilitate refolding of amyloids into monomers. The ability to control these biochemical processes could potentially lead to an effective treatment of amyloidosis family of diseases, most notably Alzheimer’s disease.^17–19^

Aqueous IL mixtures are typically employed in biochemical applications, as water is an integral part of any life-related system and assists in reducing the notoriously high viscosity of the ILs. The Hofmeister series of ions is often called in to explain the effect of the ILs on the proteins, but the conclusions seem to be protein-dependent and even contradictory.^12,20^ It is mandatory to use a wide range of experimental techniques to investigate these complex three-component protein–IL–water systems.^21,22^ For example, it has been recently demonstrated that some standard experimental techniques may provide invalid conclusions regarding interactions between green fluorescent protein (GFP) and ions of some imidazolium or pyrrolidinium ILs.^23^ UV absorption and small-angle X-ray scattering results led to the conclusion that IL did not alter the tertiary structure of GFP, and circular dichroism measurements suggested that IL did not have any effect on its secondary structure either. Only the nuclear magnetic resonance (NMR) measurements employing an advanced saturation transfer difference (STD) technique have revealed unambiguously the condensation of the ions of the IL around GFP. Even more, NMR spectroscopy allowed mapping of the interactions between individual residues of the protein with the specific ions of the IL, showing that more than 40 residues of GFP were interacting with the ions directly. These important results not only emphasize the mandate to use different complementary experimental methods to arrive at unequivocal conclusions regarding protein–IL interactions but also showcase the capabilities of NMR spectroscopy in this field. Modern STD-NMR experiments are now increasingly used to study the interactions between ILs and proteins.^24^

A viable route toward a comprehensive understanding of the molecular mechanisms behind the protein–IL interactions would be to study the behavior of close relatives of proteins—peptides and oligopeptides—in IL-based solutions, both experimentally^25–27^ and computationally.^28–33^ Like proteins, peptides are composed of proteinogenic amino acids, but their small size allows using methods which greatly aid in understanding their interactions with the ions, which are otherwise unfeasible for larger proteins. In this regard, recent experimental NMR studies of small tetra- and pentapeptides in aqueous mixtures of some imidazolium ILs are particularly informative.^34,35^ It was shown that ^1^H NMR chemical shifts of several investigated peptides exhibit prominent changes when they are dissolved in IL/water mixtures as compared to their pure aqueous solutions. The NMR chemical shifts of protons are sensitive probes of their local environment, and even changes by as little as a few hundredths of ppm are considered to be significant enough to indicate the occurrence of structural perturbations.^36^ For the tetra- and pentapeptides studied in refs 34,35 , chemical shifts of their individual protons were found in many cases to change by as much as 0.2 ppm, and in some extreme cases even by 0.5 ppm. Still, these studies employed samples with relatively high peptide concentrations, usually approaching 100 mM, implying that differential effects of interpeptide interactions in aqueous vs aqueous/IL solutions may exert a non-negligible influence on the observed differences in the recorded ^1^H NMR chemical shifts.

Aiming to dissect the nature of intermolecular interactions formed between peptides and constituent ions of an IL, we consider in this work a special case of a model tetrapeptide Ala-Glu-Pro-Phe (AEPF) in an aqueous mixture of 1-ethyl-3-methylimidazolium trifluoroacetate ([EMim]­[TFA]), see Figure 1. The AEPF peptide includes a proline residue, and a cis–trans isomerization via rotation around its N–C bond is a slow process that might generally be a rate-limiting step in protein folding^37^ and tune the biological activity of proteins.^38–41^ The AEPF peptide augmented with succinate and *p*-nitroanilide groups is in fact often used as a convenient substrate for evaluating reaction rates and activity of the peptidyl-prolyl cis–trans isomerase Pin1.^42,43^ Indeed, the AEPF peptide undergoes a very slow isomerization in solution, leading to the presence of two long-lived isomers trans- and cis-AEPF.^35^ Therefore, separate sets of ^1^H NMR signals from each of the two isomers were resolved experimentally, both in aqueous and aqueous IL solutions of the AEPF peptide.^35^

**Figure 1:**
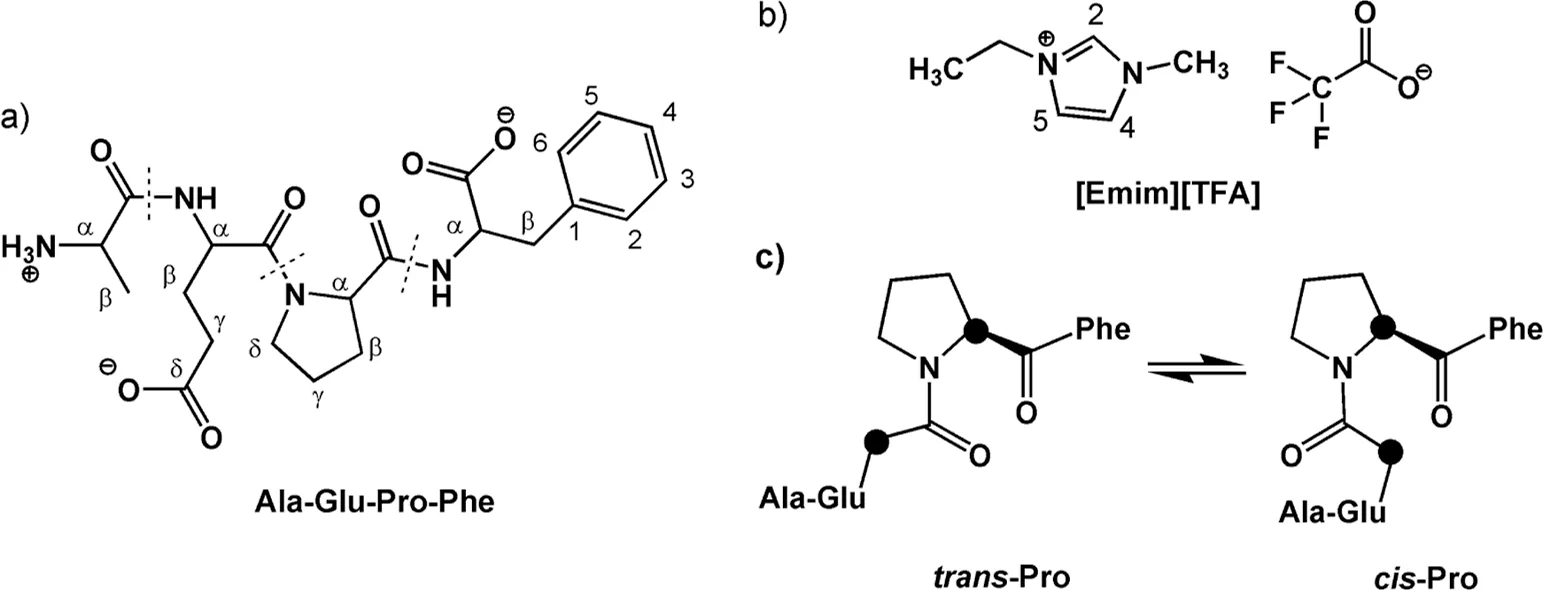
Structural formulas of tetrapeptide Ala-Glu-Pro-Phe (a) and 1-ethyl-3-methylimidazolium trifluoroacetate (b); cis–trans isomerism of Ala-Glu-Pro-Phe (c).

To exclude the possibility of interpeptide interactions completely, we consider in this work trans- and cis-AEPF peptides in an aqueous [EMim]­[TFA] mixture as well as in pure aqueous solution at infinite dilution conditions. We rely on classical molecular dynamics (MD) simulations to sample the phase spaces of molecular systems under investigation over extended simulation times on the order of hundreds of nanoseconds. Furthermore, we use combined quantum mechanics/molecular mechanics (QM/MM) approaches to compute the ^1^H NMR chemical shifts of the AEPF peptides in the condensed phase. Computational methods based on MD simulations and combined quantum-classical models for calculations of spectroscopic NMR properties have already provided an invaluable insight into structural organization of IL materials.^44–49^ Very recently, we have successfully used this computational scheme to elucidate the hydrotropic activity of choline tryptophanate IL toward aqueous solubilization of antidiabetic agent glibenclamide.^50^ Here, we hope to obtain well-motivated conclusions regarding the behavior of this small peptide in aqueous IL media. The outcome of this work is expected to contribute to general understanding of interactions between proteins and ionic liquids.

## 2 Methods

### 2.1 Molecular Dynamics Simulations

The trans- and cis-AEPF peptides were simulated in aqueous and aqueous [EMim]­[TFA] solutions. For each system, four independent MD simulation runs were carried out starting from different initial conformations of the peptide, resulting in 16 MD simulation runs in total. The aqueous IL mixture was composed of 200 [EMim]­[TFA] ion pairs and 800 H_2_O molecules, following experimental conditions implemented in ref 35. The anionic state of the Glu residue in AEPF was assumed in all systems, along with the protonated and deprotonated states of the N- and C-termini, respectively, because, under the neutral pH conditions employed in this study, the N-terminal amino group is expected to remain protonated, whereas the carboxyl groups at the C-terminus and in the side chain of Glu stay deprotonated. The total charge of the entire tetrapeptide was equal to −1. Aqueous solutions of AEPF consisted of 1 peptide, 2000 H_2_O molecules, and a Na^+^ counterion to maintain electrostatic neutrality. An aqueous [EMim]­[TFA] solution of a single trans- or cis-AEPF peptide was electrostatically neutralized by an additional EMim^+^ cation.

Force field parameters from refs 51,52 were used for EMim^+^ cations and TFA^–^ anions, respectively. Parameters from the FF14SB force field^53^ were selected for the AEPF tetrapeptide. The TIP3P model^54^ was used for water molecules, and force field parameters of Joung and Cheatham were applied for the Na^+^ counterion.^55^ The Antechamber tool^56^ of the AMBER20 suite of programs was used to assign atomic types and force field parameters. Initial molecular configurations for each system were constructed using the Packmol program.^57^ All MD simulations have been conducted using the AMBER20 program.^58^

The leapfrog algorithm with an integration time step of 1 fs was selected to solve equations of motion along with periodic boundary conditions. The Ewald summation method was adopted to treat long-range electrostatic and van der Waals interactions with the cutoff distance set to 16 Å. The Berendsen barostat^59^ was used to control the pressure with a relaxation time of 3.0 ps. The Langevin thermostat^60–62^ was utilized to maintain constant temperature with a collision frequency of 3.0 ps^–1^. Lorentz–Berthelot mixing rules were used to determine Lennard–Jones parameters for pairs of unlike atom types. Strengths of the intramolecular 1–4 electrostatic and van der Waals interactions were reduced by dividing by 2.0 and 1.2, respectively, which are the default values of these scaling factors in the Amber force field. The SETTLE algorithm^63^ was used to eliminate stretching modes of all chemical bonds involving hydrogen atoms, and the RATTLE scheme^64^ was employed to maintain the rigidity of water molecules.

MD simulations were performed at a temperature of 303 K in agreement with experimental conditions in ref 35. Every system was first subjected to an energy-minimization run to remove possible bad contacts. Initial MD simulations in the *NPT* ensemble were conducted for 10 ns at a constant pressure of 101.3 kPa, leading to the converged values of the density for every system, as can be seen in Figures S1–S10 of the Supporting Information (SI) file. Simulations were continued in the *NVT* ensemble. After equilibration for 10 ns, the production run followed for 400 ns, sampling the molecular configurations at regular intervals of 1 ps. Selected molecular trajectories were used for structural analysis, and reduced sets of 100 molecular configurations were considered in the QM/MM calculations.

### 2.2 QM/MM Calculations

To predict the ^1^H NMR isotropic shielding constants of the AEPF peptide in the condensed phase, we rely on the explicit QM/MM model.^65^ The linear-response QM/MM model based on density functional theory (DFT) and gauge-including atomic orbital approach for the calculation of the NMR magnetic shielding tensors is described in refs 66,67 and implemented in the Dalton2019 electronic structure program.^68^ The NMR shielding constants of the AEPF peptide were calculated using the PBE0 exchange–correlation functional^69^ and the Ahlrichs def2-TZVP basis set.^70,71^ To refine the model, solvent molecules with at least one atom within 3.5 Å distance from the oxygen atoms as well as within 4 Å distance from any other heavy atom of the peptide were promoted to the QM-described region of the model. For AEPF solution in aqueous [EMim]­[TFA], the cutoff distance for inclusion of solvent molecules to the QM region was set to 3.5 Å from all heavy atoms. The QM/MM calculations for the AEPF peptide in aqueous solution included 40–64 water molecules in the QM part. For peptides in aqueous IL mixtures, the QM part included 8–26 water molecules, 3–13 EMim^+^ cations, and 1–9 TFA^–^ anions. Further details concerning the composition of the QM region in the QM/MM calculations are provided in Table S1 of the SI. A small 3–21G basis set^72,73^ was used for the solvent molecules promoted to the QM region, a compromise between cost and accuracy which has previously proven to be reasonable.^45,46,50^ The rest of the solvent molecules within 20 Å radius around the center of mass of the peptide were described by the point-charge potential using the same charges as in the MD simulations.^51^ The input files for QM/MM calculations were prepared using a locally produced Python script Whirlpool 1.14. The QM/MM calculations were performed for 100 molecular configurations of each of the peptide conformers, spaced at regular intervals of at least 1.5 ns. The magnetic shielding constants of AEPF peptides in the liquid state were evaluated as arithmetic averages over all molecular configurations, and statistical errors were calculated as the standard deviation of the sample.

## 3 Results and Discussion

### 3.1 Structural Analysis

#### 3.1.1 Conformational Analysis

For each of the four systems—trans- and cis-AEPF peptides in aqueous and aqueous IL solutions—four independent MD simulation runs have been conducted, starting from a distinct conformational state of the tetrapeptide. Major conformations of AEPF originate from the rotations around chemical bonds in the backbone of the peptide. Therefore, the conformations of the AEPF tetrapeptide will be in this work classified according to the values of the Ramachandran angles φ and ψ, which indicate the torsional angles C–N–C_α_–C and N–C_α_–C–N along the backbone of the peptide chain, respectively. Furthermore, Ramachandran angles for the terminal Ala and Phe residues correspond to relatively rapid reorientations of small molecular fragments in solution, meaning that truly distinct conformations of the AEPF peptide are due to the different values of the φ and ψ angles of Glu and Pro residues only (vide infra). The four initial conformations of the peptide are thus distinguished by the φ and ψ angles of Glu and Pro residues corresponding to either polyproline-II, P_II_, or δ region in the Ramachandran diagram.^74^ The four runs are labeled as Run1 to Run4, corresponding to the following initial conformations of the peptide: Run1—(Glu P_II_, Pro δ); Run2—(Glu P_II_, Pro P_II_); Run3—(Glu δ, Pro δ); and Run4—(Glu δ, Pro P_II_).

Of the two proline isomers of the AEPF peptide, trans-AEPF with the experimentally estimated population of 81% is the dominating form in aqueous solution.^35^ Addition of [EMim]­[TFA] IL leads to further stabilization of the trans-AEPF isomer as its population rises to 85% in the aqueous IL solution of AEPF in a 70% IL/30% D_2_O mixture.^35^ The analysis of our MD trajectories obtained from 16 independent simulations, each 400 ns long, has revealed that isomeric shift between the two forms of AEPF has never occurred, as expected considering the very slow interconversion rate between the trans and cis isomers of the AEPF peptide.

To inspect the conformational spaces of the AEPF peptide, we show in Figure 2 the Ramachandran plots for trans- and cis-AEPF isomers sampled in aqueous solution. For either isomer of AEPF in aqueous solution, qualitatively similar results for Ramachandran diagrams have been obtained from all four MD simulation runs, and those shown in Figure 2 are based on the data sampled from a single run selected out of four. The most frequent conformation of the trans-AEPF peptide in aqueous solution corresponds to the P_II_ region of both Glu and Pro residues of the peptide. Analysis of all four trajectories sampled for trans-AEPF in aqueous solution allows estimating the populations of the P_II_ and β-sheet regions of the Glu residue to be around 80 and 20%, respectively. The population of the P_II_ region of the Pro residue is found to be around 75%, while that of the δ region accounts for the remaining 25%. No correlations between the conformational states of Glu and Pro residues were found. We also note the high interconversion rates between conformations corresponding to P_II_ and β-sheet regions of Glu and P_II_ and δ regions of Pro residues, with dozens of interconversions observed over 400 ns production runs, as can be seen in Figures S11 and S12 of the SI.

**Figure 2:**
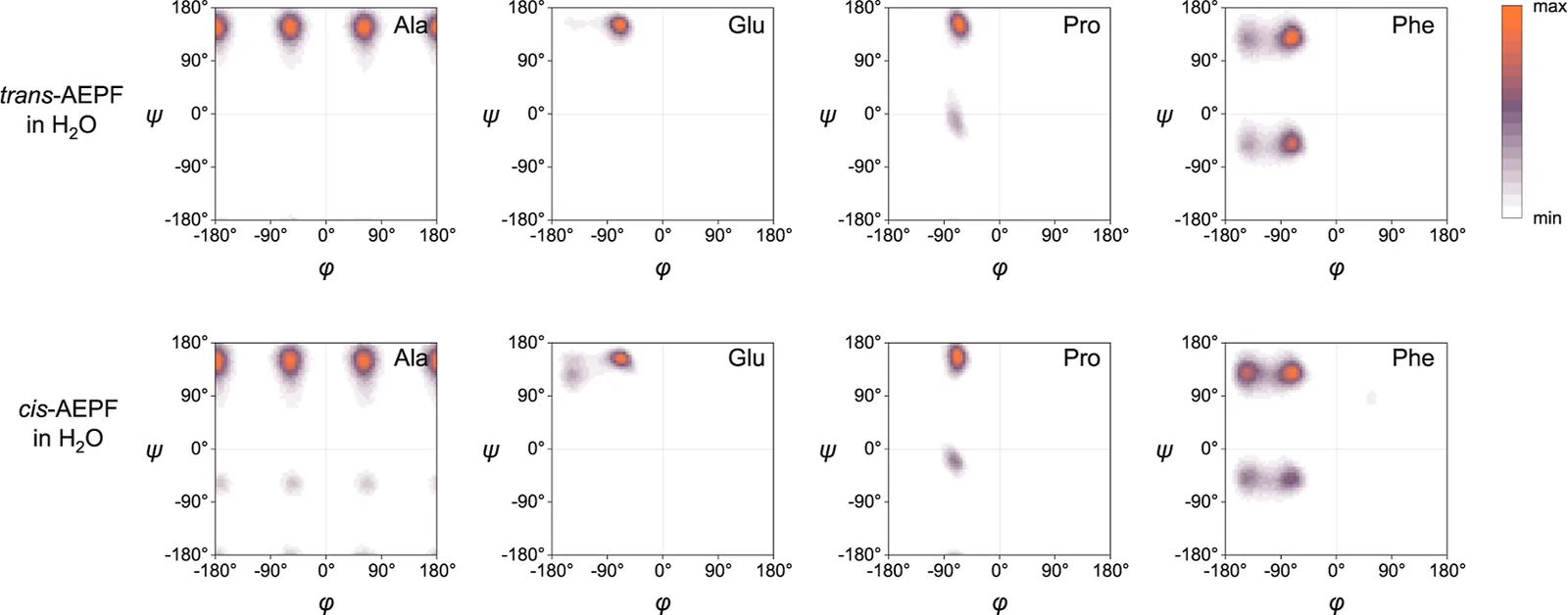
Residue-specific Ramachandran diagrams for trans (top panel) and cis (bottom panel) proline isomers of the AEPF peptide in aqueous solution. The color chart indicates the qualitative range of angular distributions—white (zero count) to red (highest count).

Similar conformational distribution in terms of the Ramachandran diagram was also found for cis-AEPF in aqueous solution, see bottom panel of Figure 2. Compared to trans-AEPF in aqueous solution, the most notable differences originate in the conformational distribution of the Glu residue. The population of the P_II_ region now amounts to around 55%, and—in addition to the population of the β-sheet region of around 42%—a small population of the δ′ region with ϕ ∼45° and ψ ∼60° of around 3% has appeared. The interconversion rate between the conformations of the Glu residue remained high just as seen for the trans-AEPF isomer in aqueous solution, see Figure S13 of the SI file. The P_II_ and δ regions are still the two common conformational states for the Pro residue. However, the energy barrier for the interconversion between the two has become noticeably higher for the cis-AEPF isomer as just a few interconversion events were observed on the course of 400 ns only, as exemplified in Figure S14 of the SI. The populations of the P_II_ and δ conformational states of the Pro residue vary from around, respectively, 68 and 32% to 93 and 7% among the four runs, with average values of around 78 and 22%, respectively. Clearly, the duration of the simulations of 400 ns is too short to reliably quantify the conformational distribution of the Pro residue of the cis-AEPF peptide in aqueous solution.

The analysis of the Ramachandran diagrams of terminal Ala and Phe residues in Figure 2 reveals rapid, almost unhindered rotations of the terminal NH_3_^+^ group in Ala and the terminal carboxylate moiety in the Phe residue occurring in aqueous solution. The most probable values of the ψ angle of Ala are found to be in the range of around 140°–160°, demonstrating the preference of the NH_3_^+^ group of Ala to mostly stay on the same side as its carbonyl group. Distribution of the φ angle in the relatively wide region of around −160° to –50° signifies conformational flexibility of the Phe residue in aqueous solution of the AEPF peptide in terms of the rotation around its N–C bond.

The Ramachandran diagrams of Glu and Pro residues sampled for the trans-AEPF isomer in aqueous [EMim]­[TFA] IL solution are shown in Figure 3. We note that the Ramachandran plots sampled for Ala and Phe residues for trans- as well as cis-AEPF isomer in aqueous IL solution are reminiscent of those recorded in aqueous solution, as shown in Figure 2, meaning that the conformational distributions of these terminal residues are qualitatively the same in either solvent. Therefore, they will not be discussed in the following. It is seen in Figure 3 that four MD simulation runs executed for the trans-AEPF peptide in aqueous IL solution lead to rather distinct Ramachandran plots. The primary reason for this is the increased viscosity of the aqueous [EMim]­[TFA] mixture and therefore slowed-down dynamics of the system. The analysis of the Ramachandran diagrams in Figure 3 allows identifying four major conformations of the trans-AEPF isomer in aqueous IL solution. These diagrams hint that the conformation with the populated P_II_ region of both Glu and Pro residues might be the most frequent conformation of AEPF in aqueous IL solution, i.e., the same conformation as in aqueous solution. However, pronounced populations of other three conformations with Glu and Pro in the regions of P_II_ and δ have still been observed. Particularly peculiar is the case of the conformation with the Glu residue being in the δ and Pro being in the P_II_ region, see Figure 3d. Here, the conformation of the peptide has never changed neither during the equilibration phase nor during the production run of 400 ns.

**Figure 3:**
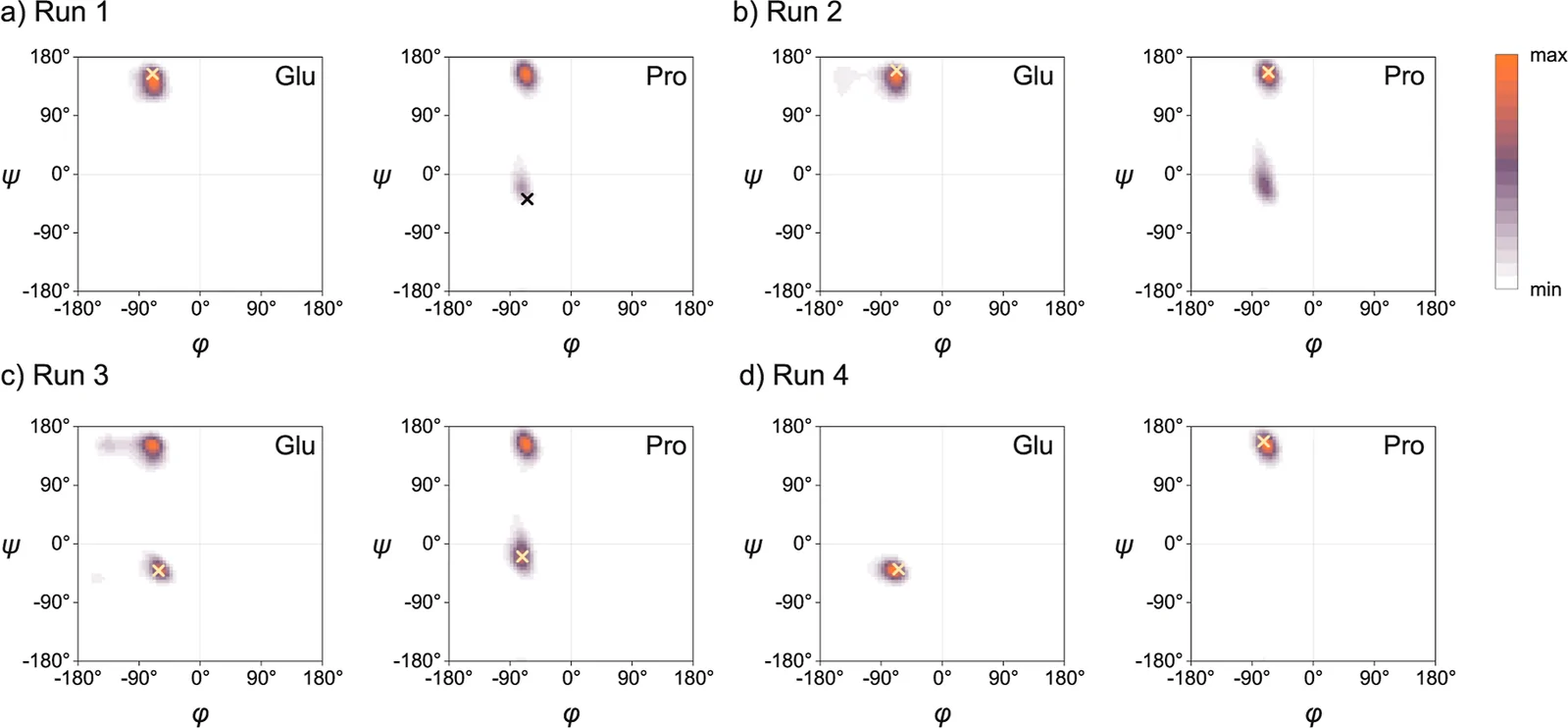
Ramachandran diagrams for residues Glu and Pro in the trans-AEPF peptide in aqueous [EMim]­[TFA] solution sampled during four independent MD simulations labeled Run1 to Run4. The crosses in the diagrams indicate the initial conformational state of Glu and Phe residues in each run. The color chart indicates the qualitative range of angle distributions—white (zero count) to red (highest count).

The Ramachandran diagrams of Glu and Pro residues sampled for the cis-AEPF isomer in aqueous [EMim]­[TFA] IL solution are shown in Figure 4. The results in Figure 4a,b allow identifying the conformation of AEPF with both the Glu and Pro residues in the P_II_ region as the most abundant conformation of the cis-AEPF peptide in aqueous IL solution. However, just as in the case of the trans-AEPF isomer, the conformation of the peptide with the Glu residue being in the δ and Pro being in the P_II_ region is found to be persistent again. The Run3 series of MD simulation indicates a pronounced population of the conformation with Glu and Pro residues both being in the δ region, see Figure 4c. Furthermore, a rather unique conformation of the peptide with the Glu residue sampling the β-sheet region has also been observed in Run3.

**Figure 4:**
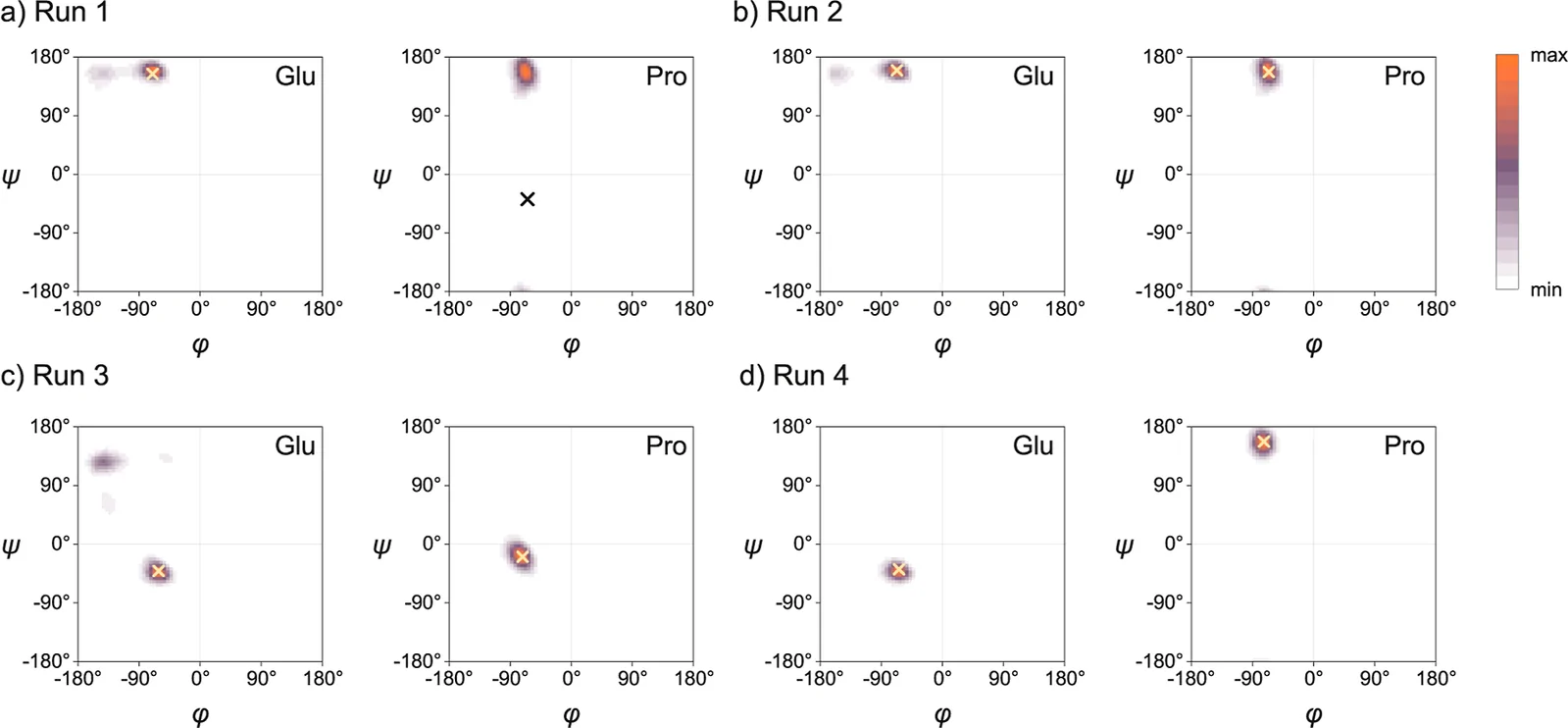
Ramachandran diagrams for residues Glu and Pro in the cis-AEPF peptide in aqueous [EMim]­[TFA] solution sampled during four independent MD simulations labeled Run1 to Run4. The crosses in the diagrams indicate the initial conformational state of Glu and Phe residues in each run. The color chart indicates the qualitative range of angle distributions—white (zero count) to red (highest count).

The structural results in Figures 3 and 4 indicate clearly that the duration of the MD simulation even as long as 400 ns for the AEPF in aqueous [EMim]­[TFA] solution is too short to obtain converged results for the conformational distribution of the AEPF tetrapeptide, which is the result of the much increased viscosity of this mixture as compared to that of aqueous solution. This finding is particularly evident from the recorded time evolutions of the Ramachandran angles for the Glu and Pro residues in trans- and cis-AEPF isomers shown in Figures S15–S22 and S23–S30, respectively, in the SI file, where in most cases just a few conformational interconversion events were observed. The results of the present study thus do not allow for reliable quantitative prediction of the conformational equilibrium of AEPF in an aqueous IL solution. It is apparent that simulations on the scale of at least microseconds or perhaps even tens of microseconds would be needed to achieve this goal. However, we assume that all important conformations of the peptide have been met in present MD simulations for sufficiently long periods of time, so that equilibrium configurational spaces of solvent distribution around these conformers have been properly sampled. Therefore, we will proceed to discuss the structural and later spectroscopic NMR results for each major conformation of the peptide in an aqueous IL solution separately. The immediate advantage to be gained by such an approach is due to the enhanced level of systematicity as we are in this way obtaining the conformational resolution of structural and spectroscopic data discussed hereafter.

In Figure 5, geometrical features of the major conformational states of the two isomers of the AEPF tetrapeptide are illustrated.

**Figure 5:**
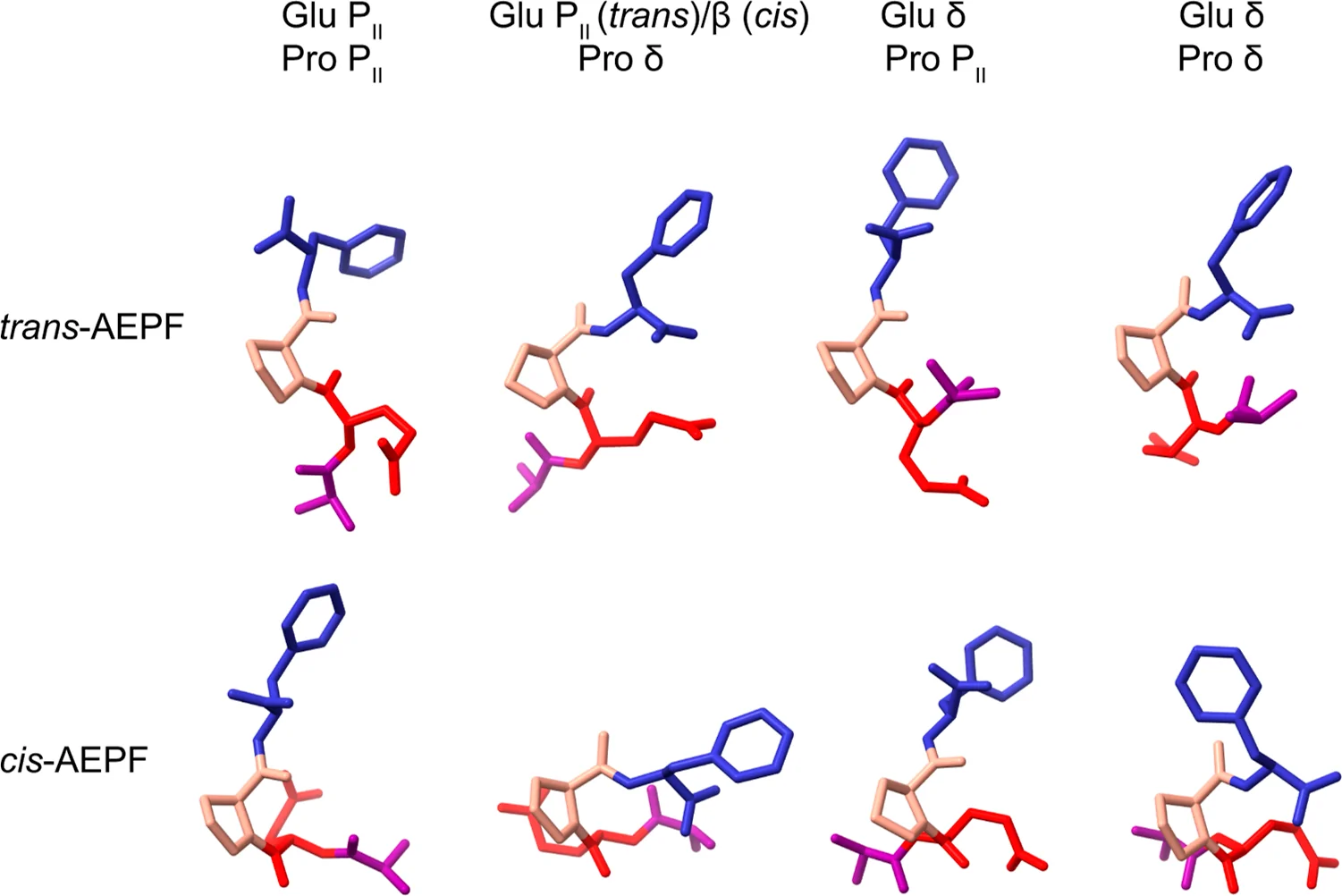
Representative structures of conformers of trans- and cis-AEPF tetrapeptides observed in aqueous and aqueous [EMim]­[TFA] solutions. The Ala, Glu, Pro, and Phe residues are colored in magenta, red, pink, and blue, respectively. Hydrogen atoms have been stripped for clarity.

It is easy to recognize that the geometries of the trans isomer are mostly extended, whereas those of the cis isomer present more compact structures with the Ala–Glu fragment being closer to the Phe residue. Intramolecular hydrogen bonding is seen to assist in maintaining some of these conformations. In particular, the hydrogen bond between the terminal NH_3_^+^ group of Ala and the COO^–^ group of Phe was seen to be virtually always formed for the conformation of the cis-AEPF peptide with Glu and Pro residues corresponding to the β-sheet and δ states, respectively. The same intramolecular hydrogen bond has been observed for the conformation of the trans-AEPF peptide with Glu and Pro residues both corresponding to the δ state, where this interaction was seen to persist for a duration of around 40 ns. In addition, the hydrogen bonding between the N–H group of Phe and the carbonyl oxygen atom of the Ala residue was also found to contribute to the stability of this closed conformation of the trans-AEPF peptide. Otherwise, the side chain of the Glu residue was seen to be rather dynamic in both aqueous and aqueous IL solutions, forming and disrupting the intramolecular hydrogen bond with either the N–H group of Glu, the NH_3_^+^ group of Ala, or the N–H group of the Phe residue. These intramolecular hydrogen bonds were found to be occasionally formed in all conformational states of the AEPF tetrapeptide with the exception of the already mentioned closed conformation of the cis-AEPF with the Glu residue in the β-sheet and Pro in the δ states.

#### 3.1.2 Solvation Structure

The analysis of the structural distribution of water molecules and constituent ions of [EMim]­[TFA] IL around AEPF in aqueous IL solution has been performed for each of the four distinct long-lived conformers of either isomer of the tetrapeptide separately. As discussed above, these conformers are distinguished by the conformational states of Glu and Pro residues. For the trans-AEPF isomer, the backbone conformations of the Glu and Pro residues correspond to the following regions of the Ramachandran diagram: (Glu P_II_, Pro P_II_), (Glu P_II_, Pro δ), (Glu δ, Pro P_II_), and (Glu δ, Pro δ). The same conformational states have been sampled for the cis-AEPF isomer as well, just the (Glu P_II_, Pro δ) conformer has been replaced by the (Glu β, Pro δ) conformation instead. The structural analysis was performed for continuous 100 ns long portions of the trajectories, where each individual conformation of the peptide was seen to be present uninterruptedly. In the cases with longer trajectories available for specific conformers of the peptide, our testing has revealed that structural analysis of the 100 ns long trajectory is sufficient to achieve converged results.

The structure of the solvation shell of each conformer of the trans- and cis-AEPF peptides in aqueous IL solution will be gauged against that seen for the respective isomer in aqueous solution. For the trans-AEPF peptide in aqueous solution, structural analysis was performed on a randomly selected 100 ns long portion of the trajectory. However, as discussed above, the equilibrium conformational populations of the Pro residue of cis-AEPF in an aqueous solution could not be determined in a reliable manner. For this reason, we have resorted to performing the structural analysis of aqueous solvation around the cis-AEPF peptide for its conformation where the Pro residue is exclusively in the P_II_ region, i.e., for the dominating conformational state of the cis-AEPF peptide in aqueous solution.

To inspect how the introduction of [EMim]­[TFA] IL alters the structure of the aqueous solvation shell of AEPF, we have calculated the coordination numbers of water molecules around all heavy atoms of the peptide in aqueous as well as aqueous IL solution. These coordination numbers were calculated by spherical integration of the specific radial distribution functions up to the distance of 4 Å. The differences in the coordination numbers of water molecules between the two solvents for trans- and cis-AEPF peptides are shown in Figures 6 and 7, respectively. As expected, coordination numbers of water molecules around virtually every atom of the peptide have become smaller in aqueous IL solution as compared to those in aqueous solution. General profiles of the curves seen in Figures 6 and 7 are rather similar, and they do not differ much neither among the two proline isomers, nor among the distinct conformations. It is apparent that the depletion of the solvation shell of the peptide by water molecules is most pronounced around solvent exposed portions of the peptide. This includes the N- and C-termini of the peptide and the side chains of the Glu, Pro, and Phe residues. The smallest change in the amount of water molecules in the first solvation shell were thus observed for the N–C_α_ atoms in the backbone of Glu, Pro, and Phe residues because of the steric hindrance.

**Figure 6:**
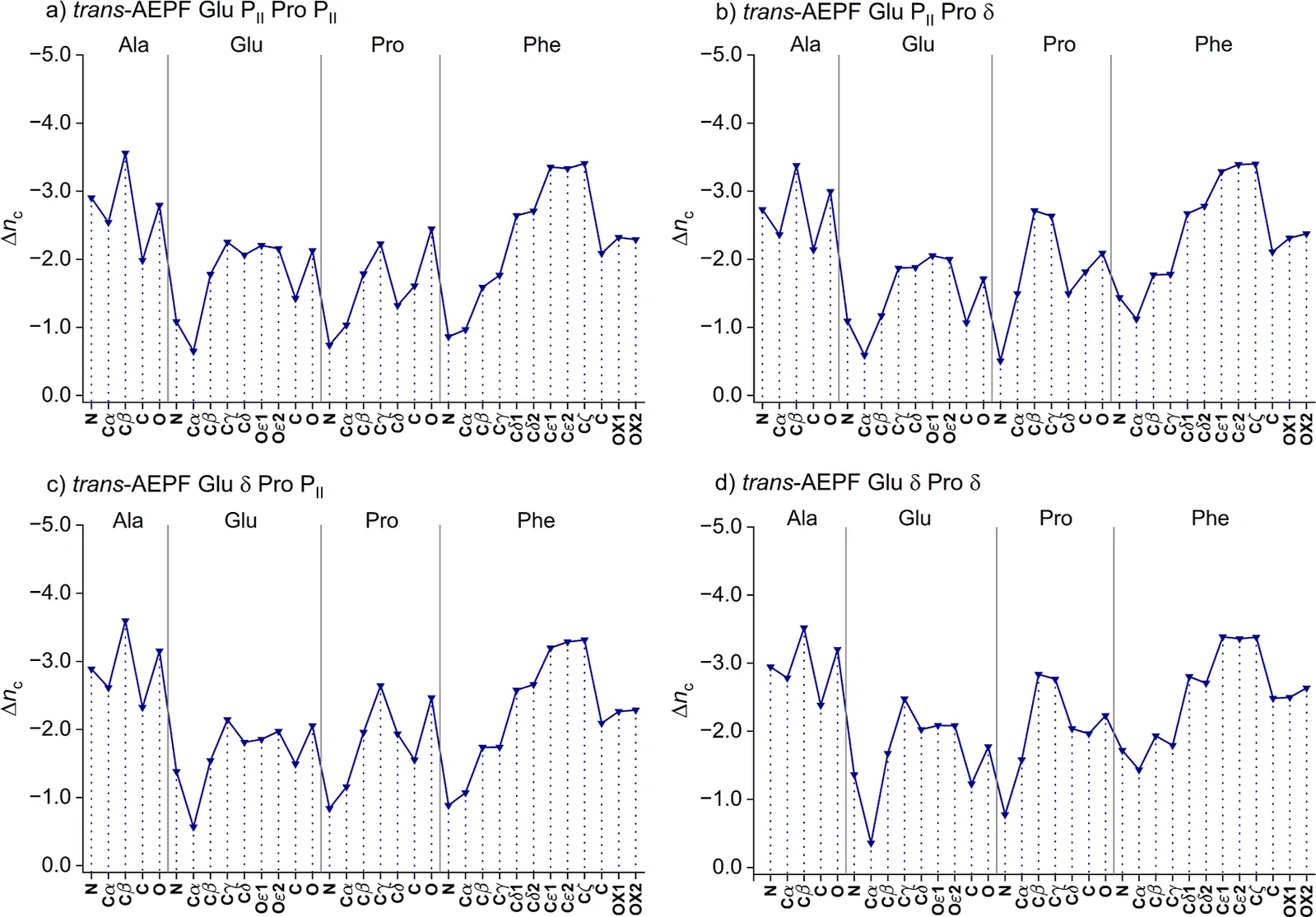
Differences between the coordination numbers of water molecules around the heavy atoms of trans-AEPF between aqueous IL and aqueous solutions, Δ*n*_c_, for four distinct conformational states of the peptide.

**Figure 7:**
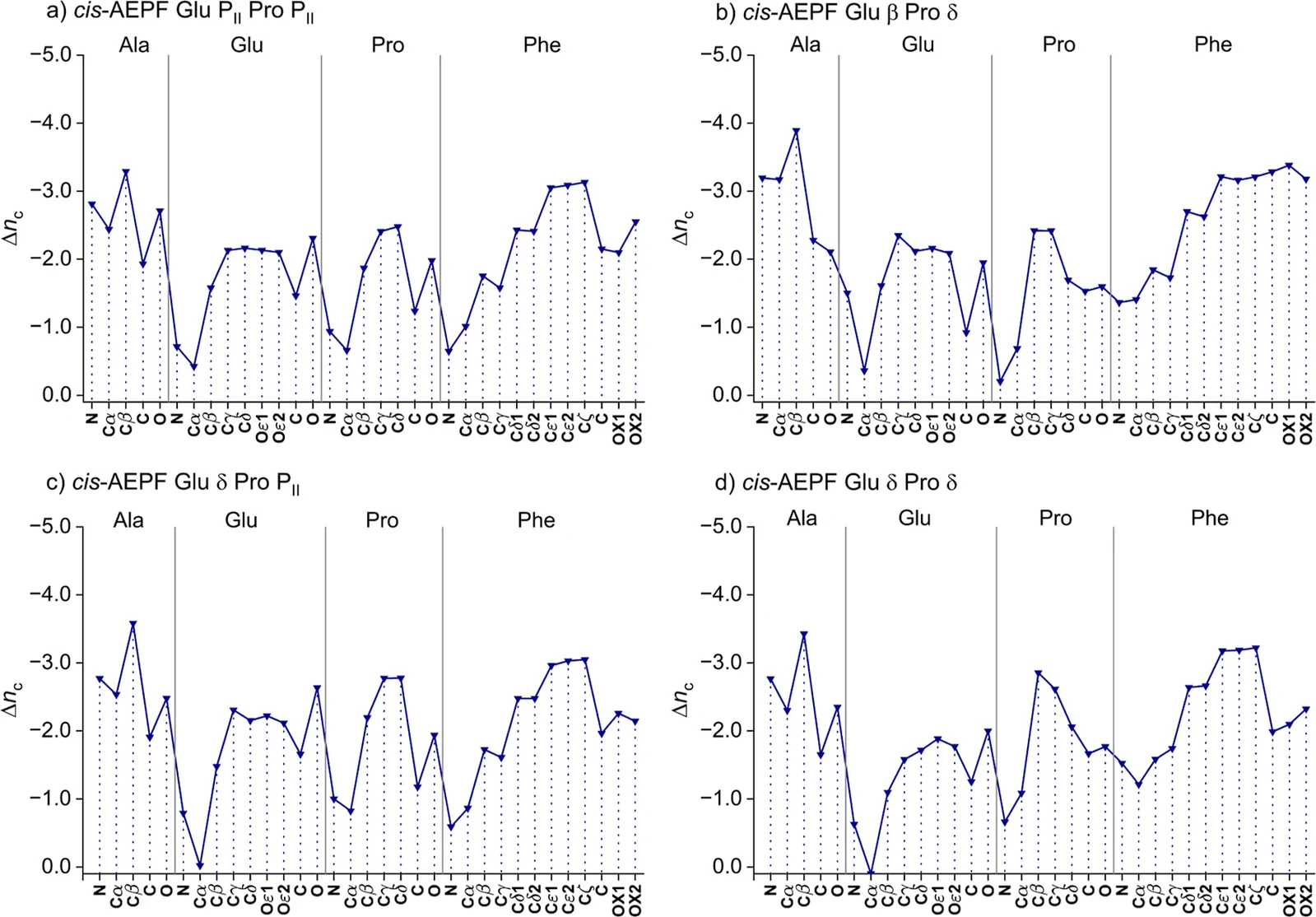
Differences between the coordination numbers of water molecules around the heavy atoms of cis-AEPF between aqueous IL and aqueous solutions, Δ*n*_c_, for four distinct conformational states of the peptide.

In Figures 8 and 9, we show the coordination numbers of EMim^+^ cations and TFA^–^ anions around the heavy atoms of the trans- and cis-AEPF peptides in aqueous [EMim]­[TFA]­solution. These coordination numbers were computed by integrating the radial distribution functions calculated between heavy atoms of the peptide and the centers of mass of the atoms in the imidazolium ring of EMim^+^ cations or the midpoints of the C–C bond in TFA^–^ anions up to the distance of 5.5 Å. It is generally seen that both the cations and anions of the [EMim]­[TFA] IL are to be met in the first solvation shell of the peptide. However, EMim^+^ cations have generally greater affinity of condensing around the peptide, as particularly evident in the case of the most stable (Glu P_II_, Pro P_II_) conformation of the trans-AEPF peptide, see Figure 8a. An obvious exception is the N-terminus of the peptide where hydrogen bonding between its NH_3_^+^ group and the carboxylate moieties of the TFA^–^ anions is particularly pronounced. The Glu residue having an anionic carboxylate group at the end of the side chain is naturally mostly solvated by the EMim^+^ cations. Meanwhile, the environment of the Pro residue is found to be the least specific for the type of ion as cations and anions were found to be in the vicinity of the Pro residue with more or less similar probability. The Phe residue is clearly more favorably solvated by the EMim^+^ cations which is a consequence of hydrogen bonding between the terminal carboxylate moieties and the EMim^+^ cations, as well as of possible stacking interactions between the benzene ring of Phe and the imidazolium rings of EMim^+^ cations. Generally, however, the pattern of solvation of the peptide by ions of the IL is seen to be little dependent on the isomer or the conformational state of the peptide.

**Figure 8:**
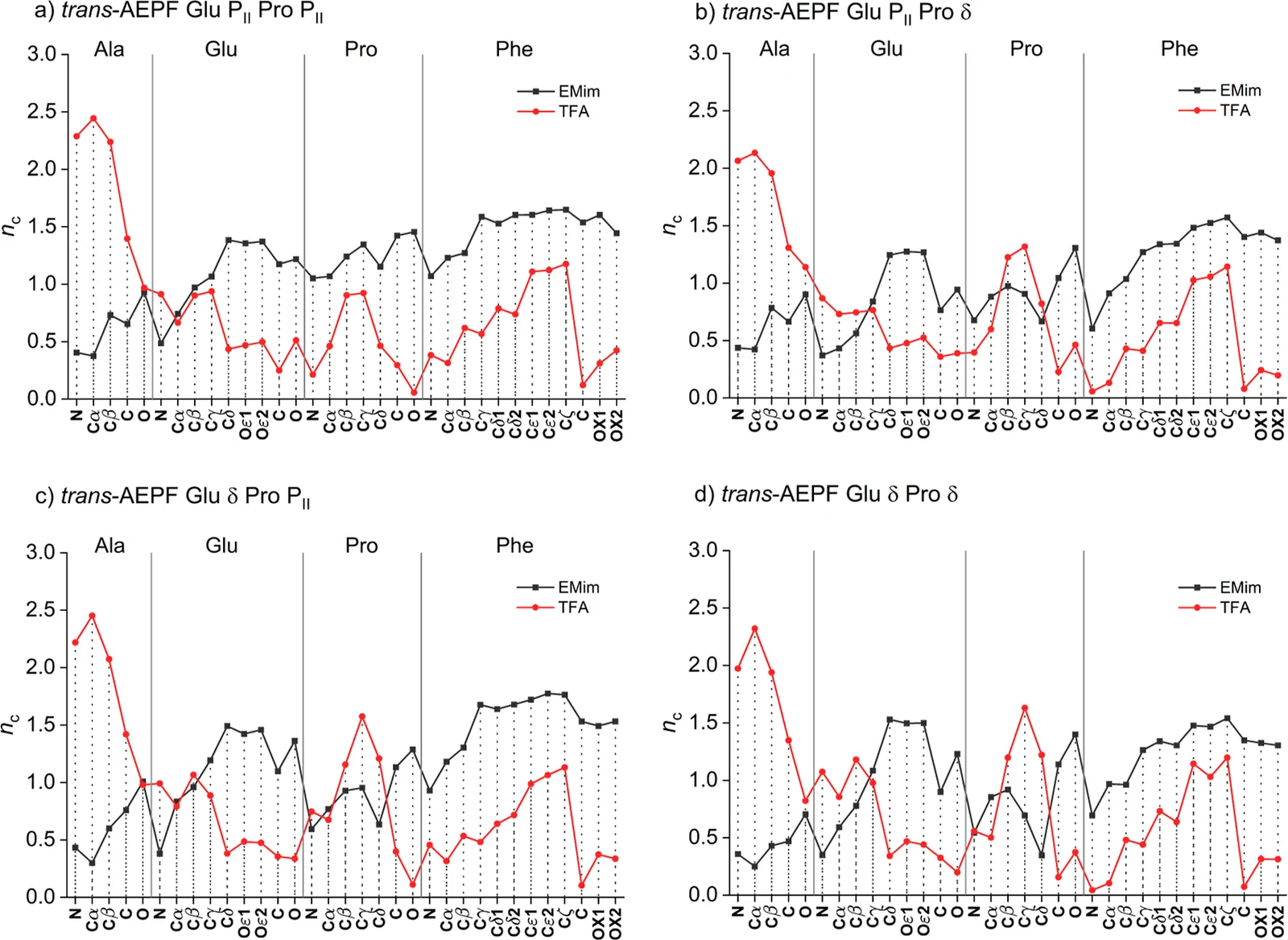
Coordination numbers of EMim^+^ cations and TFA^–^ anions around the heavy atoms of trans-AEPF in aqueous IL solution, *n*_c_, for four distinct conformational states of the peptide.

**Figure 9:**
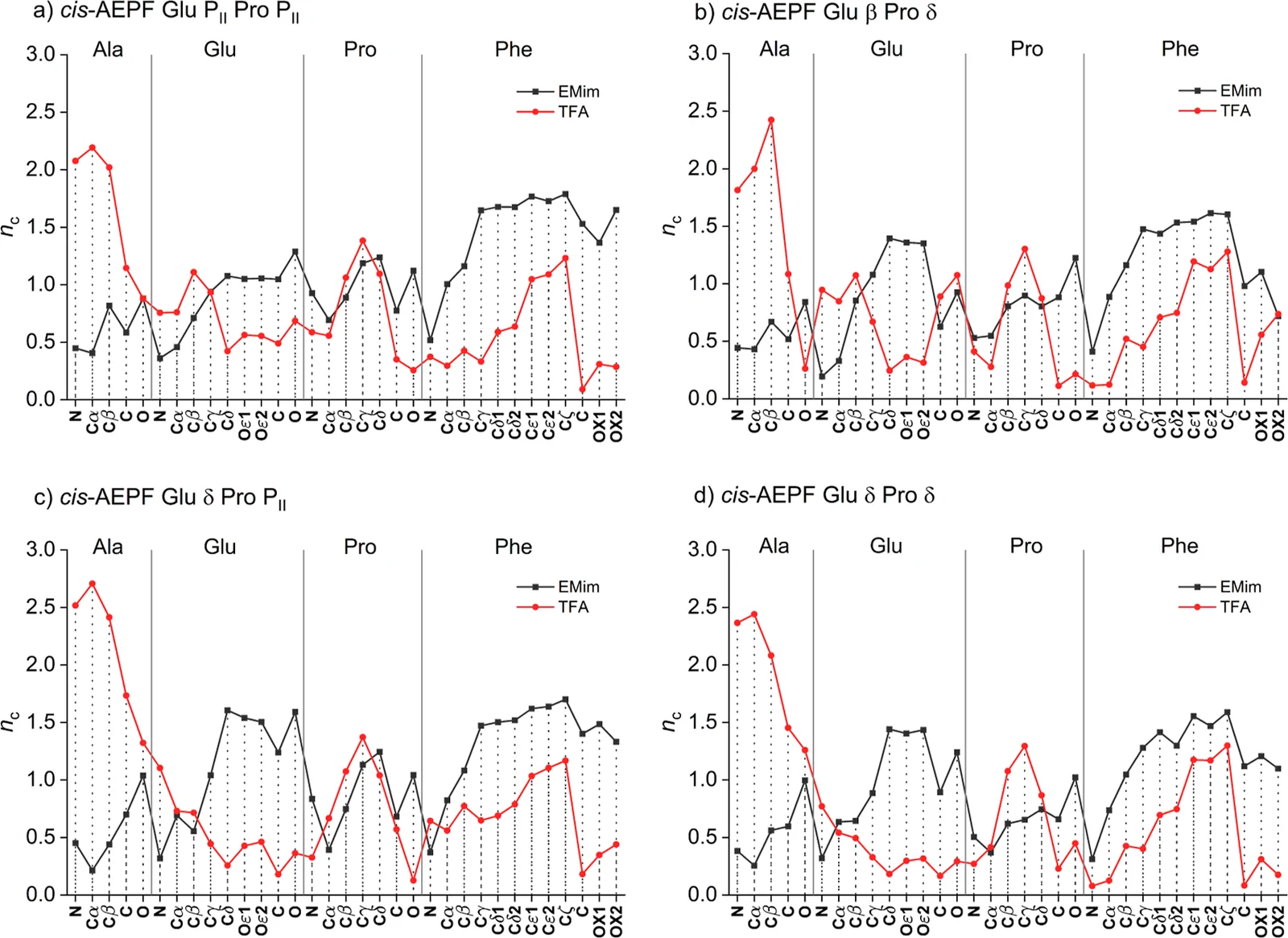
Coordination numbers of EMim^+^ cations and TFA^–^ anions around the heavy atoms of cis-AEPF in aqueous IL solution, *n*_c_, for four distinct conformational states of the peptide.

The AEPF peptide is capable of forming hydrogen bonds with the constituent ions of [EMim]­[TFA], both as a donor and as an acceptor. However, TFA^–^ anions possess only hydrogen-bond-accepting property, while the EMim^+^ cations may only act as hydrogen bond donors through the C–H bonds at positions 2, 4, and 5 in the imidazolium ring,^75^ see Figure 1b. In Table 1, the coordination numbers of the oxygen atoms in the TFA^–^ anions around the hydrogen-bond-donating nitrogen atoms of the trans- and cis-AEPF peptide conformations are presented. Unsurprisingly, the TFA^–^ anions form prominent hydrogen bonding interactions with the NH_3_^+^ moiety at the N-terminus of the peptide, and the corresponding coordination numbers seen in Table 1 show only a slight variation with the conformation of either isomer of AEPF. The N–H group of the Glu residue in the backbone of the peptide is fairly frequently involved in hydrogen bonding with the anions, while that of the Phe residue shows the smallest affinity for hydrogen bonding. This is particularly true for the closed conformations of the peptide where the access for the TFA^–^ anions to the N–H group in the backbone of Phe is restricted sterically. Under these circumstances, the coordination numbers were found to be plainly equal to zero. It is generally seen that the extent of hydrogen bonding between the AEPF and the TFA^–^ anions is little dependent on both the isomeric and conformational states of the peptide.

**Table 1:** Coordination Numbers of Hydrogen-Bond-Accepting Oxygen Atoms in TFA^–^ Anions around the Hydrogen-Bond-Donor Sites in the AEPF Peptide in Aqueous [EMim]­[TFA] Solution^a^

isomer	conformer	Ala NH_3_^+^	Glu NH	Phe NH
trans-AEPF	Glu P_II_, Pro P_II_	2.46	0.61	0.24
	Glu P_II_, Pro δ	2.18	0.46	0.00
	Glu δ, Pro P_II_	2.46	0.65	0.28
	Glu δ, Pro δ	2.39	0.67	0.00
cis-AEPF	Glu P_II_, Pro P_II_	2.29	0.54	0.21
	Glu β, Pro δ	2.23	0.87	0.00
	Glu δ, Pro P_II_	2.66	0.71	0.53
	Glu δ, Pro δ	2.63	0.43	0.00

a All values of coordination numbers have been computed by spherical integration of the respective N­(AEPF)···O­(TFA^–^) radial distribution functions up to 3.375 Å.

The coordination numbers of the three hydrogen atoms in the imidazolium ring of EMim^+^ cations around the hydrogen-bond-accepting oxygen atom sites of the peptide are collected in Table 2. The two oxygen atoms of the carboxylate moieties in the Glu or Phe residue are equivalent, and the values of the coordination numbers provided in Table 2 have been in these instances computed around one of the oxygen atoms. The most hydrogen-bond-prone site of AEPF is its terminal carboxylate group. The EMim^+^ cations attack this site most frequently through their most acidic C–H group at position 2, although the C_4_–C_5_ edge of the imidazolium ring is seen to approach the terminal carboxylate group fairly frequently as well. The coordination numbers show minor variation with the conformation of the peptide, yet the smallest values were computed for the (Glu δ, Pro δ) conformation of trans-AEPF and (Glu β, Pro δ) conformation of the cis-AEPF peptide. These are the closed conformations of the peptide with prominent intramolecular hydrogen bonding between peptides’ N- and C-termini. The C_2_–H_2_ group of EMim^+^ cations is clearly the most frequent hydrogen bond partner also for the carboxylate group in the side chain of the Glu residue. Due to the flexibility of this side chain, the competition between intramolecular and intermolecular hydrogen bonding involving the carboxylate moiety of Glu is seen to take place. The smallest values for the coordination numbers of the hydrogen atoms of EMim^+^ cations around the carboxylate of the Glu residue were found in those instances where the intramolecular hydrogen bonding with this particular group of the peptide was the most prolific.

**Table 2:** Coordination Numbers of Hydrogen-Bond-Donor Atoms in EMim^+^ Cations around the Hydrogen-Bond-Accepting Sites in the AEPF Peptide in Aqueous [EMim]­[TFA] Solution^a^

isomer	conformer		Ala O	Glu O	Glu Oε	Pro O	Phe O
trans-AEPF	Glu P_II_, Pro P_II_	H_2_	0.19	0.35	0.47	0.54	0.75
		H_4_	0.22	0.25	0.18	0.25	0.23
		H_5_	0.26	0.21	0.19	0.19	0.14
	Glu P_II_, Pro δ	H_2_	0.23	0.17	0.43	0.22	0.61
		H_4_	0.21	0.22	0.19	0.31	0.22
		H_5_	0.18	0.15	0.16	0.26	0.18
	Glu δ, Pro P_II_	H_2_	0.52	0.37	0.45	0.38	0.69
		H_4_	0.14	0.27	0.19	0.24	0.22
		H_5_	0.09	0.20	0.16	0.17	0.14
	Glu δ, Pro δ	H_2_	0.22	0.28	0.53	0.27	0.57
		H_4_	0.11	0.21	0.20	0.25	0.20
		H_5_	0.08	0.21	0.17	0.24	0.14
cis-AEPF	Glu P_II_, Pro P_II_	H_2_	0.25	0.22	0.32	0.22	0.66
		H_4_	0.19	0.30	0.15	0.23	0.18
		H_5_	0.14	0.22	0.12	0.17	0.12
	Glu β, Pro δ	H_2_	0.22	0.22	0.50	0.30	0.44
		H_4_	0.19	0.17	0.17	0.25	0.19
		H_5_	0.15	0.17	0.14	0.20	0.16
	Glu δ, Pro P_II_	H_2_	0.36	0.45	0.67	0.21	0.76
		H_4_	0.24	0.27	0.18	0.20	0.21
		H_5_	0.17	0.25	0.17	0.17	0.16
	Glu δ, Pro δ	H_2_	0.26	0.30	0.48	0.20	0.55
		H_4_	0.25	0.24	0.17	0.23	0.15
		H_5_	0.20	0.19	0.16	0.24	0.12

a All values of coordination numbers have been computed by spherical integration of the respective O­(AEPF)···H_X_(EMim^+^), X = 2, 4, 5, radial distribution functions up to 3.375 Å. See Figure 1b for atom labeling in EMim^+^ cations.

The access to the carbonyl groups in the backbone of the peptide is sterically restricted for the anions, unlike that for the solvent-exposed carboxylate moieties. Consequently, the coordination numbers of the EMim^+^ cations around the oxygen atoms of the carbonyl groups in Ala, Glu, and Pro residues are considerably smaller in most cases as compared to those seen around the solvent-exposed carboxylate groups of the peptide. Furthermore, all three C–H groups of the imidazolium ring are mostly seen to form hydrogen bonds with more or less equal probability. Overall, we have observed a significant formation of hydrogen bonding interactions between the peptide and the EMim^+^ cations of the IL. The AEPF peptide offers more hydrogen-bond-accepting rather than donating sites, and this might be one of the reasons why EMim^+^ cations were seen to displace water molecules around the peptide more effectively than the TFA^–^ anions.

Another reason for this comes due to the possibility of the π–π stacking interactions between the phenyl ring in the side chain of the Phe residue and the imidazolium rings of the EMim^+^ cations. The distributions of the absolute magnitude of the cosine of an angle between the planes of the benzene ring in the Phe residue and the 5-membered imidazolium ring in the EMim^+^ cations for a given distance between the two rings are shown in Figure 10. As seen in this figure, the formation of π–π stacking between the two types of rings is evident as the angular distribution shows pronounced maxima with the absolute magnitude of cos θ close to 1 for the distances between the two rings in the range of around 3.6–4.1 Å. We have performed the statistical analysis to quantify the extent of π–π stacking interactions for each conformer of either isomer of AEPF by using a conventional geometrical criterion for stacked configuration with the distance between the centers of the rings of not higher than 4.5 Å and the angle between their planes not exceeding 30°. This analysis has revealed that the prevalence of π–π stacking interactions is similar among four considered conformations of the trans-AEPF isomer, occurring from around 48% of configurations for (Glu P_II_, Pro δ) and (Glu δ, Pro δ) conformations up to around 56% for the (Glu δ, Pro P_II_) conformation. The average number of EMim^+^ cations forming the stacked configurations with the phenyl ring of the Phe residue ranges from 1.12 to 1.19 and thus is fairly similar among the four conformers of trans-AEPF. The phenyl ring is therefore mostly found in stacked configuration with a single EMim^+^ cation and only occasionally sandwiched between two cations. Similar results were found for the cis-AEPF isomer, although here the (Glu P_II_, Pro P_II_) conformation was found to exhibit the highest affinity for π–π stacking interactions as these were found to be present in around 68% of configurations over the course of 100 ns. For this conformation of the cis-AEPF peptide, the probability for two EMim^+^ cations to form a stacked configuration with the phenyl ring of Phe is also relatively the highest among all of the conformations of either trans- or cis-AEPF peptide. The extent of π–π stacking is similar among other three conformations of the cis-AEPF peptide, ranging from around 48 to 56%. Figure S31 of the SI provides a representative illustration of specific hydrogen bonding and π–π stacking interactions formed between the AEPF tetrapeptide and the EMim^+^ and TFA^–^ ions in solution.

**Figure 10:**
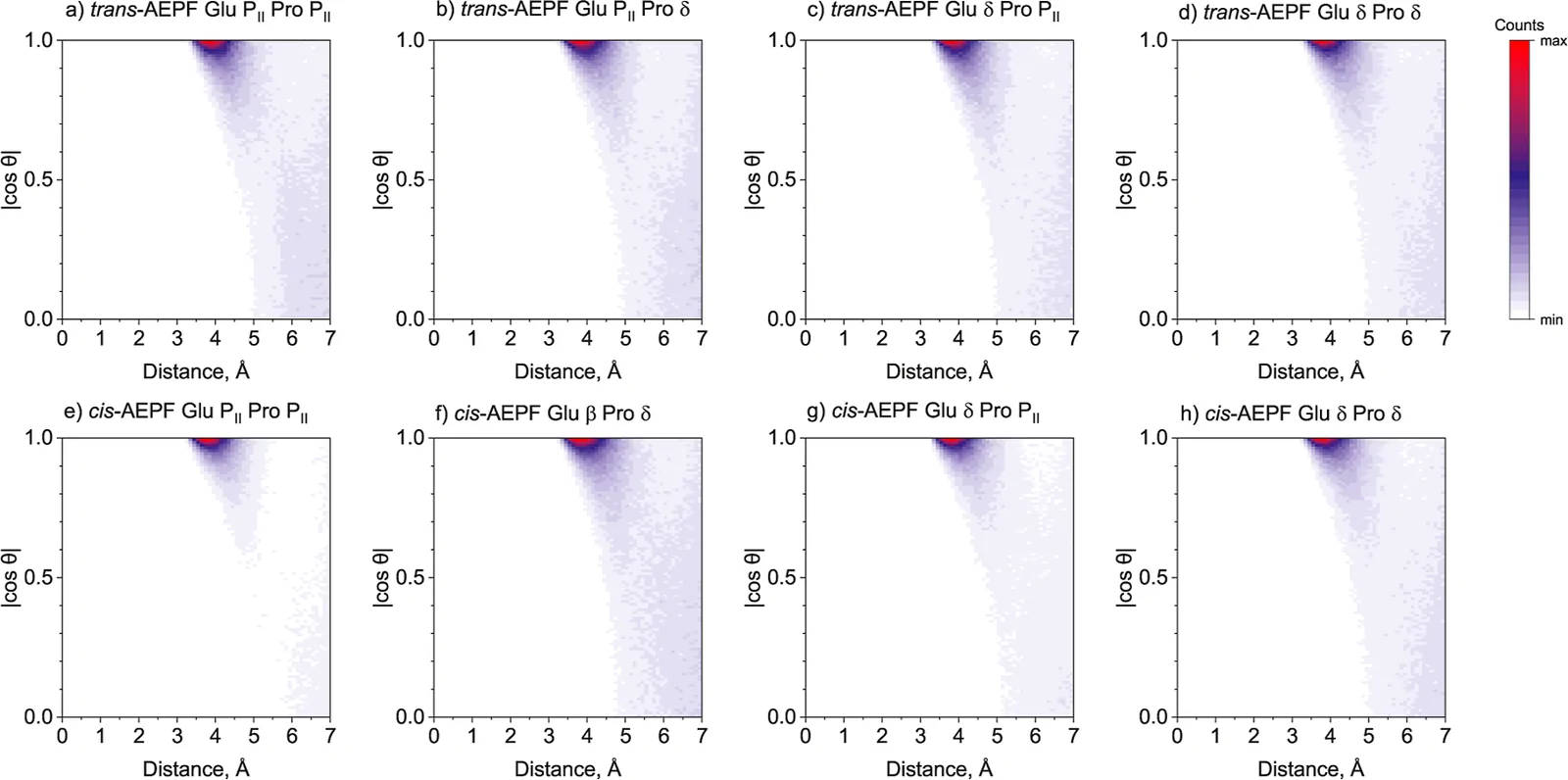
Distribution of the magnitude of cosine of an angle between the planes of the benzene ring in the Phe residue of AEPF and the imidazolium ring in the EMim^+^ cations, θ, for a given distance between the centers of mass of the two rings. Distributions have been computed for each conformation of the peptide separately, and they are shown in the top and bottom panels for the trans- and cis-AEPF isomers, respectively.

### 3.2 ^1^H NMR Chemical Shifts

NMR measurements on AEPF peptides carried out in ref 35 have revealed that their ^1^H NMR spectra recorded in aqueous solution and in an aqueous [EMim]­[TFA] mixture are generally different, suggesting that spectral changes occur due to the direct interactions of the peptide with the constituent ions of the IL. However, it is important to bear in mind a very high concentration of the peptide used in the samples in this study, reaching 90 mM. Under these circumstances, the effect of peptide self-aggregation on the recorded NMR data cannot be excluded, and therefore, the direct comparison between the experimental findings of ref 35 and our computational results performed under the conditions of infinite dilution of the peptide is hardly possible. Still, it is instructive to observe that—as illustrated in Figure 2 of ref 35—the changes in the ^1^H NMR chemical shifts of trans- and cis-AEPF going from aqueous solution to aqueous IL solution were seen to be rather similar in both qualitative and quantitative terms, despite structural differences between the two proline isomers of the peptide. The ^1^H NMR signals of Ala and Glu residues were found to be the most sensitive to the introduction of the IL into aqueous solution, while those of the Pro residue were seen to be affected less as the changes of the chemical shifts did not exceed 0.1 ppm. The signals of the protons in the phenyl ring of the Phe residue were the only ones experiencing prominent upfield shifts upon introduction of the IL. In virtually all other cases, downfield shifts of the ^1^H NMR absorption peaks of AEPF were recorded.

We have performed extensive DFT-based QM/MM calculations of the ^1^H NMR isotropic shielding constants of trans- and cis-AEPF peptides in aqueous and aqueous [EMim]­[TFA] solutions. The entire first solvation shell of the peptide has been promoted to the QM region of the model, and the point-charge potential was applied for solvent molecules beyond that. On the basis of the computed NMR isotropic shielding constants, we have evaluated how the ^1^H NMR signals of individual protons in four different conformers of both the trans- and cis-AEPF peptides shift when going from aqueous solution to aqueous IL solution. The changes in the NMR chemical shifts of the two isomers of AEPF were evaluated w.r.t. those computed for the respective isomer in aqueous solution, i.e., employing the same references as those in evaluating the differences in the distribution of water molecules around the peptide between aqueous and aqueous IL environments, as discussed in Section 3.1.2.

Computational NMR results for the four distinct conformations of the trans-AEPF peptide are illustrated in Figure 11. The chemical shift of the protons in the terminal NH_3_^+^ group in the trans-AEPF isomer was found to undergo a sizable downfield shift of up to 0.4 ppm which is to be expected considering significant presence of the hydrogen-bonded TFA^–^ anions around this group in aqueous IL solution. However, the chemical shifts of the protons at positions α and β in the Ala residue were predicted to be little affected by the change in the solvent, at most increasing by around 0.2 ppm. For all four conformations of trans-AEPF, a very large downfield shift of the NMR signals of the proton in the N–H group of the Glu residue was computed which is in line with the observed proclivity of this group to be involved in hydrogen bonding with the TFA^–^ anions. As the N–H group of Glu is apparently in a slow-exchange regime in both aqueous and aqueous IL solutions, the ^1^H NMR signal of this proton was observed in ref 35 and displayed a similarly strong downfield shift in aqueous [EMim]­[TFA] solution. The rest of the ^1^H NMR signals of the Glu residue were seen to experience marginal alterations, again of up to ±0.2 ppm, for the four conformations of the trans-AEPF peptide.

**Figure 11:**
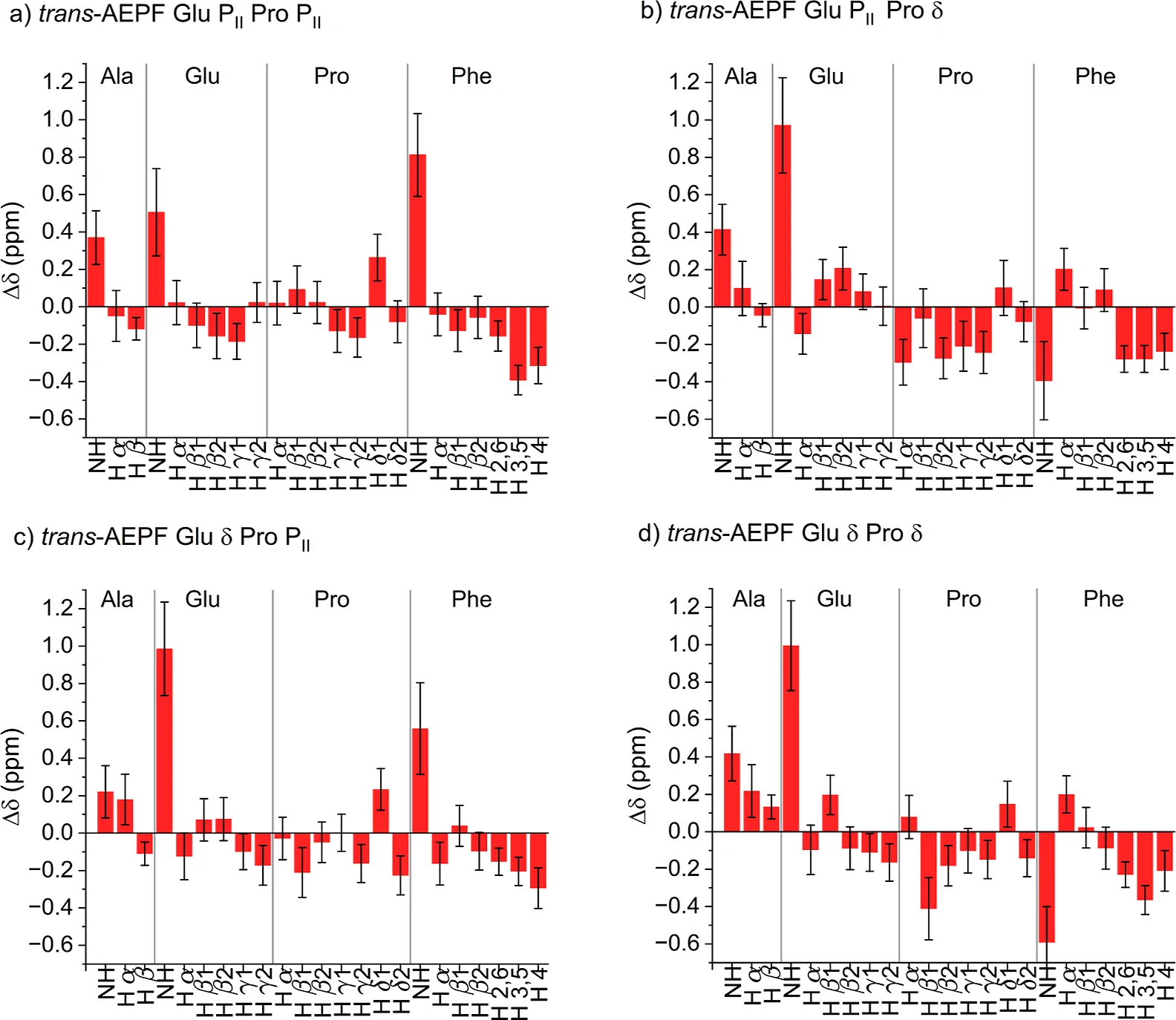
QM/MM-based aqueous-to-aqueous [EMim]­[TFA] solution changes in ^1^H NMR chemical shifts of four distinct conformations of the trans-AEPF peptide, including statistical errors for each entry.

Just as for the protons in the CH_2_ groups of Glu, the ^1^H NMR chemical shifts of the Pro residue exhibit rather small changes as well. However, it is interesting to note that the chemical shifts of the protons at the β and γ positions of the proline ring show slight sensitivity to the conformational state of the peptide. For the two conformers of the peptide with the Pro residue in the δ conformation, these four NMR signals exhibited a clear trend to undergo upfield shifts, whereas they were seen to be changed considerably less and in either direction in the case of the P_II_ conformation of the Pro residue. Even more pronounced conformational dependence was found for the behavior of the chemical shift of the proton in the N–H group of the Phe residue. The chemical shift of this proton increases prominently for the two conformations of the peptide corresponding to the P_II_ state of the Pro residue. Again, this can be easily understood due to the formation of hydrogen bonds with the carboxylate moiety of the TFA^–^ anions, as discussed in Section 3.1.2. However, the access to the N–H bond of the Phe residue was seen to be restricted sterically for the TFA^–^ anions in the case of the peptide’s conformations corresponding to the δ state of the Pro residue, and in addition, the coordination by water molecules was seen to be considerably diminished in aqueous IL solution as well, see Figure 6b,d. Therefore, the NMR signal of the proton in the N–H bond of the Phe residue undergoes a pronounced upfield shift for the conformations of the peptide with the δ state of Pro.

The chemical shifts of the aromatic protons in the side chain of the Phe residue undergo upfield shifts systematically across all four conformations of the trans-AEPF peptide. This should be considered as a clear spectroscopic manifestation of established π–π stacking interactions between the phenyl ring of Phe and the EMim^+^ cations. The same spectral alterations were also recorded experimentally in ref 35, and thus, our computational results offer a solid proof that π–π stacking is truly formed between these moieties in the aqueous [EMim]­[TFA] solutions of the AEPF peptide.

The computed changes in the ^1^H NMR chemical shifts of the cis-AEPF isomer between aqueous and aqueous [EMim]­[TFA] solutions are shown in Figure 12. The behavior of the ^1^H NMR signals of protons in the Ala residue is qualitatively similar to that seen for the trans-AEPF isomer above, noting only that the changes in the chemical shifts of the protons at α and β positions exhibit somewhat larger upfield shifts for the distinctive (Glu β, Pro δ) conformation of the cis-AEPF isomer. However, the chemical shift of the proton at the α position in the Glu residue showed markedly different behavior for the two isomers of the AEPF peptide. While for the trans isomer the corresponding chemical shift was seen to be marginally affected by the introduction of the IL, in the case of the cis isomer, this chemical shift was increased significantly even by as much as ∼1 ppm. Furthermore, the chemical shift of the proton at the α position in the Pro residue was also seen to exhibit similar effect as it also undergoes a considerable downfield shift—though somewhat smaller in magnitude—in the aqueous IL solutions of the cis-AEPF peptide, while it was seen to change much less in the case of the trans-AEPF isomer. We therefore may identify these two spectral features as potential NMR markers for distinguishing the two proline isomers of the AEPF peptide. Like in the case of the trans-AEPF isomer, the chemical shifts of protons in the side chain of the Pro residue of cis-AEPF mostly exhibit upfield shifts, most notably for the (Glu δ, Pro δ) conformation.

**Figure 12:**
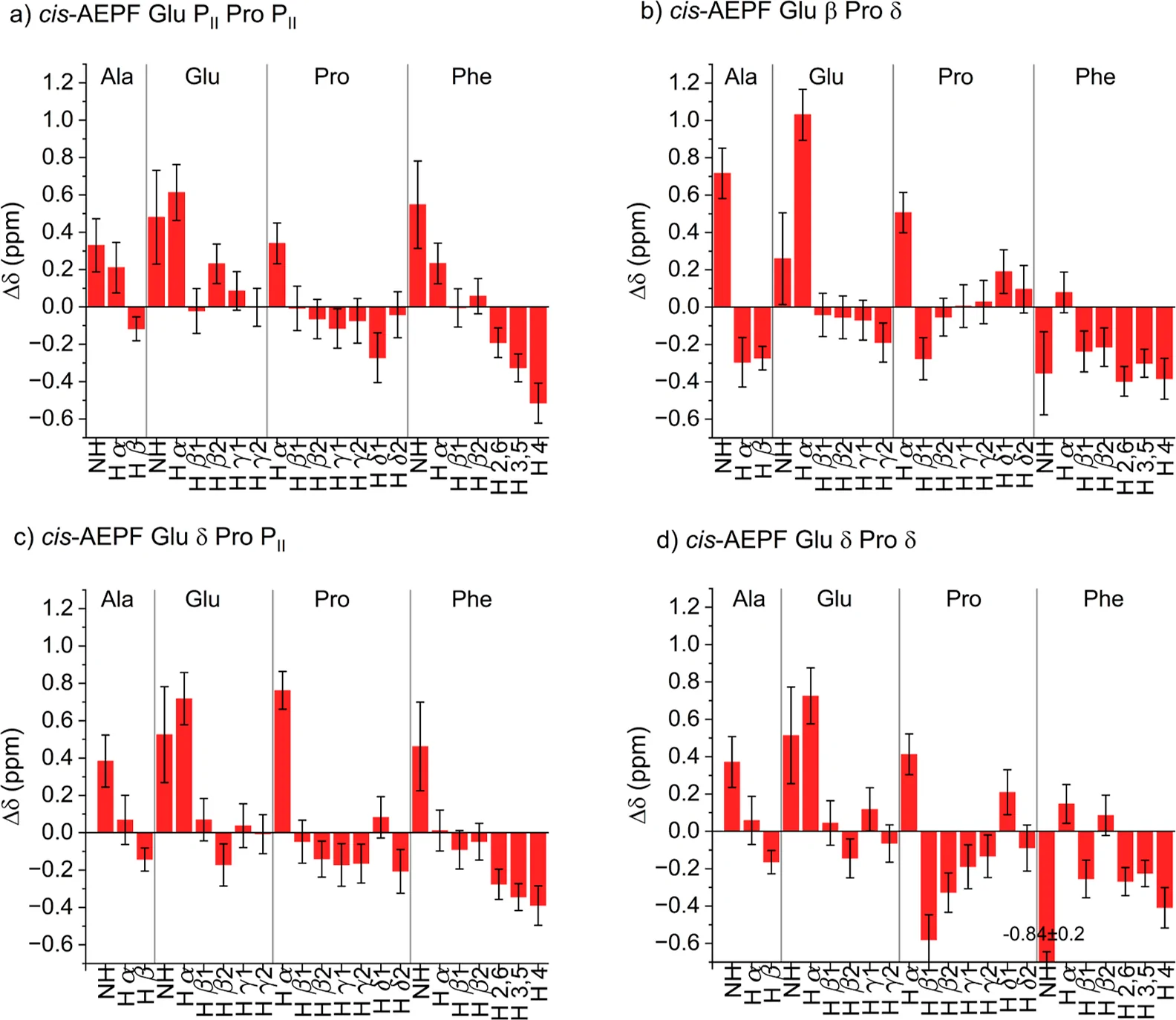
QM/MM-based aqueous-to-aqueous [EMim]­[TFA] solution changes in ^1^H NMR chemical shifts of four distinct conformations of the cis-AEPF peptide, including statistical errors for each entry.

Just as for the trans-AEPF, the chemical shift of the N–H group in the backbone of the Phe residue exhibited qualitatively similar behavior upon introduction of the IL. For the (Glu P_II_, Pro P_II_) and (Glu δ, Pro P_II_) conformations of cis-AEPF, this chemical shift was seen to move downfield, whereas for the (Glu β, Pro δ) and (Glu δ, Pro δ) conformations, it moved upfield. Again, these downfield shifts are due to the hydrogen bonding with the TFA^–^ anions and upfield shifts are due to the reduced intensity of hydrogen bonding with water molecules. It is thus interesting that the value of the ^1^H NMR chemical shift of the N–H moiety in Phe may serve as a spectroscopic marker of the conformational state of the Pro residue in the AEPF peptide, as the N–H group of Phe is apparently in a slow exchange regime in solution and thus can be observed experimentally.^35^ Finally, just as in the case of the trans-AEPF peptide, the prominent upfield shifts of the signals from aromatic protons in the phenyl ring of Phe are spectroscopic signatures of the π–π stacking with the imidazolium rings.

The findings of the present pilot study illustrate well the challenges that need to be resolved in order to achieve a sound comparison between experimental and computational NMR results for complex molecular systems such as biopolymers in three-component aqueous IL mixtures. A systematic experimental NMR study on small prototypical peptides in IL/water solutions is currently lacking, yet it would be a welcome addition opening the door for rigorous benchmarking of computational NMR methodologies. Not only ^1^H but also ^13^C and even ^15^N as well as ^17^O NMR measurements would be highly valuable, allowing to fully characterize the behavior of peptides in solution. The concentration of the peptide should be kept low enough so that peptide aggregation could be excluded. Furthermore, the dependence of NMR chemical shifts on the composition of aqueous IL mixtures should be investigated as well. While the experimental NMR findings of Richardt et al.^35^ are important in their own right, the experimental conditions implemented in their work do not offer this level of systematicity.

The reliability of computational NMR results based on the integrated scheme combining MD simulations and QM/MM methods for molecules in traditional molecular solvents has generally been well assessed. Prediction of the NMR properties for IL systems is not without challenges, but some progress has been demonstrated recently.^49,76,77^ However, it might still be appropriate to emphasize the importance of several factors that govern the accuracy of the computational scheme. Concerning classical MD simulations, the quality of the force field is of the utmost importance. While we have used a state-of-the-art AMBER force field for the peptide, the potential designed specifically for [EMim]­[TFA] IL is not available to date, and thus, force field parameters for cations and anions from different sources had to be used. Also, more elaborate interaction potentials including explicit treatment of polarization interactions may be required to sample the phase spaces of biopolymers in IL solutions in a more reliable manner.^28^ In addition, due to the high viscosity of IL systems, advanced sampling techniques might be necessary to involve in order to fully account for the conformational equilibrium of the solutes in aqueous IL solutions. This constitutes an essential prerequisite for a well-founded comparison of experimental and computational results.

Concerning electronic structure calculations of NMR shielding constants using the QM/MM model, the choice of the electronic structure method, e.g., DFT functional, and one-electron basis set is of crucial importance as well.^78,79^ Even though molecules in the first solvation shell of the peptide were treated at the DFT level along with the peptide, it was currently only feasible to apply a modest 3–21G basis for these solvent molecules. All these sources of error are in action when computing the NMR shielding constants of the peptide in both aqueous and aqueous IL solutions, and error cancellation in evaluating changes between shielding constants computed in both solutions cannot be guaranteed, nor systematically controlled, as was generally seen previously.^80^ These points will have to be considered in seeking better qualitative and quantitative agreement between computational and experimental results for the NMR spectroscopic properties of biomolecules in IL-based solutions.

## 4 Summary

In this study, the structural and NMR spectroscopic properties of an Ala-Glu-Pro-Phe tetrapeptide in aqueous and aqueous [EMim]­[TFA] mixtures were scrutinized by using an integrated computational scheme comprising classical MD simulations and linear-response QM/MM methods. MD simulations of two trans and cis proline isomers of AEPF in both types of solvents have been carried out using the AMBER force field for the peptide and IL-specific potential for [EMim]­[TFA]. Four independent MD simulation runs were carried out for either isomer starting from distinct initial conformations of the peptide, and the configurational phase spaces were sampled for 400 ns in each run. In the DFT-based QM/MM calculations of the ^1^H NMR shielding constants, the first coordination shell of the peptide was treated quantum mechanically, albeit using a smaller basis set than that applied to the solute. The NMR shielding constants of the AEPF peptides in solution were evaluated as statistical averages over sets of 100 molecular snapshots sampled during MD simulations.

MD simulations have revealed that the most abundant conformation of both proline isomers of the peptide in aqueous solution corresponds to the P_II_ state of both the Glu and Pro residues, although other minor conformations including the β-sheet state of Glu or the δ state of the Pro residue could not be ignored, especially in the case of the cis isomer. The intramolecular dynamics of the AEPF peptide in aqueous IL solution was found to be severely damped as compared to that seen in aqueous solution, preventing the possibility of quantifying the conformational equilibrium of the peptide in this viscous solvent. Four major conformations of the peptide related to the conformational states of the Glu and Pro residues were observed in the simulations, and the properties of each conformation of either isomer, sampled for at least 100 ns in solution, have been analyzed separately.

Structural analysis has generally revealed that greater affinity to condense around the peptide is displayed by the EMim^+^ cations rather than by the TFA^–^ anions. This finding could be at least partly rationalized considering that the peptide includes more hydrogen-bond-accepting sites for the EMim^+^ cations than the donating sites for the TFA^–^ anions and contains a negatively charged Glu residue. Furthermore, specific π–π stacking interactions between the phenyl ring of the Phe residue and the imidazolium rings of the EMim^+^ cations were found to be abundant as well. The structural features of the first solvation shell of the peptide in aqueous IL solution are generally similar among the two proline isomers, and only some differences were observed comparing the cases of four different conformations of the same isomer, mostly related to the hydrogen bond formation abilities of specific chemical groups.

The QM/MM computations of the ^1^H NMR chemical shifts of trans and cis isomers of the peptide have shown that the ^1^H NMR spectra in aqueous and aqueous [EMim]­[TFA] solutions are considerably different. The patterns of the IL-induced changes in the ^1^H NMR chemical shifts of the peptide were found to be mostly similar between the two isomers of the peptide and between the distinct conformations of either isomer. However, our computational results allow proposing that ^1^H NMR chemical shifts of a few specific sites of the peptide could be used as spectroscopic markers of either the conformational or isomeric state of the peptide. Finally, the QM/MM computations have shown that the NMR signals of the aromatic protons in the phenyl ring of the Phe residue undergo a considerable upfield shift upon the introduction of the [EMim]­[TFA] IL, manifesting the formation of the π–π stacking interactions and confirming available experimental findings.

## Supplementary Material



## Data Availability

The data underlying this study are available in the published article and the Supporting Information.

## Notes

Evolutions of the mass densities of all systems simulated in the *NPT* ensemble at a temperature of 303 K and a pressure of 101.3 kPa; evolutions of the Ramachandran angles of Glu and Pro residues in the AEPF peptide of all systems recorded during the production runs of 400 ns in the *NVT* ensemble; further details of the composition of the QM region in the QM/MM calculations; and a representative figure of specific intermolecular interactions between the AEPF peptide and the ions of the [EMim]­[TFA] ionic liquid (PDF)
