# Supplementary material for: Modeling Peptides in Aqueous Ionic Liquid Mixtures: A Quantum Mechanics/Molecular Dynamics Study of Structural and NMR Properties
Source: J Chem Theory Comput. 2026 Aug 1;22(16):8700–16. doi: 10.1021/acs.jctc.6c01192 (PMC13523636; doi:10.1021/acs.jctc.6c01192)
Supplement: Supplementary file 1 [file ct6c01192_si_001.pdf]

Dated: July 21, 2026

# Supporting Information for:

## Modeling Peptides in Aqueous Ionic Liquid Mixtures: a Quantum Mechanics/Molecular Dynamics Study of Structural and NMR Properties

by

Žyginta Murnikova, Kęstutis Aidas*

Institute of Chemical Physics, Faculty of Physics, Vilnius University,
Saulėtekio ave. 3, Vilnius 10257, Lithuania

*E-mail: kestutis.aidas@ff.vu.lt, Phone: +370 5 223 4593.

# Contents

**1. Convergence of density in the MD simulations** **S2**

**2. Evolution of the Ramachandran angles** **S7**
2.1 *Trans*- and *cis*-AEPF in aqueous solution . . . . . . . . . . . . . . . . . . . . . S7
2.2 *Trans*-AEPF in aqueous ionic liquid solution . . . . . . . . . . . . . . . . . . S9
2.3 *Cis*-AEPF in aqueous ionic liquid solution . . . . . . . . . . . . . . . . . . . . . S12

**3. Details of the QM/MM calculations** **S15**

**4. The pattern of specific intermolecular interactions** **S16**

# 1. Convergence of density in the MD simulations

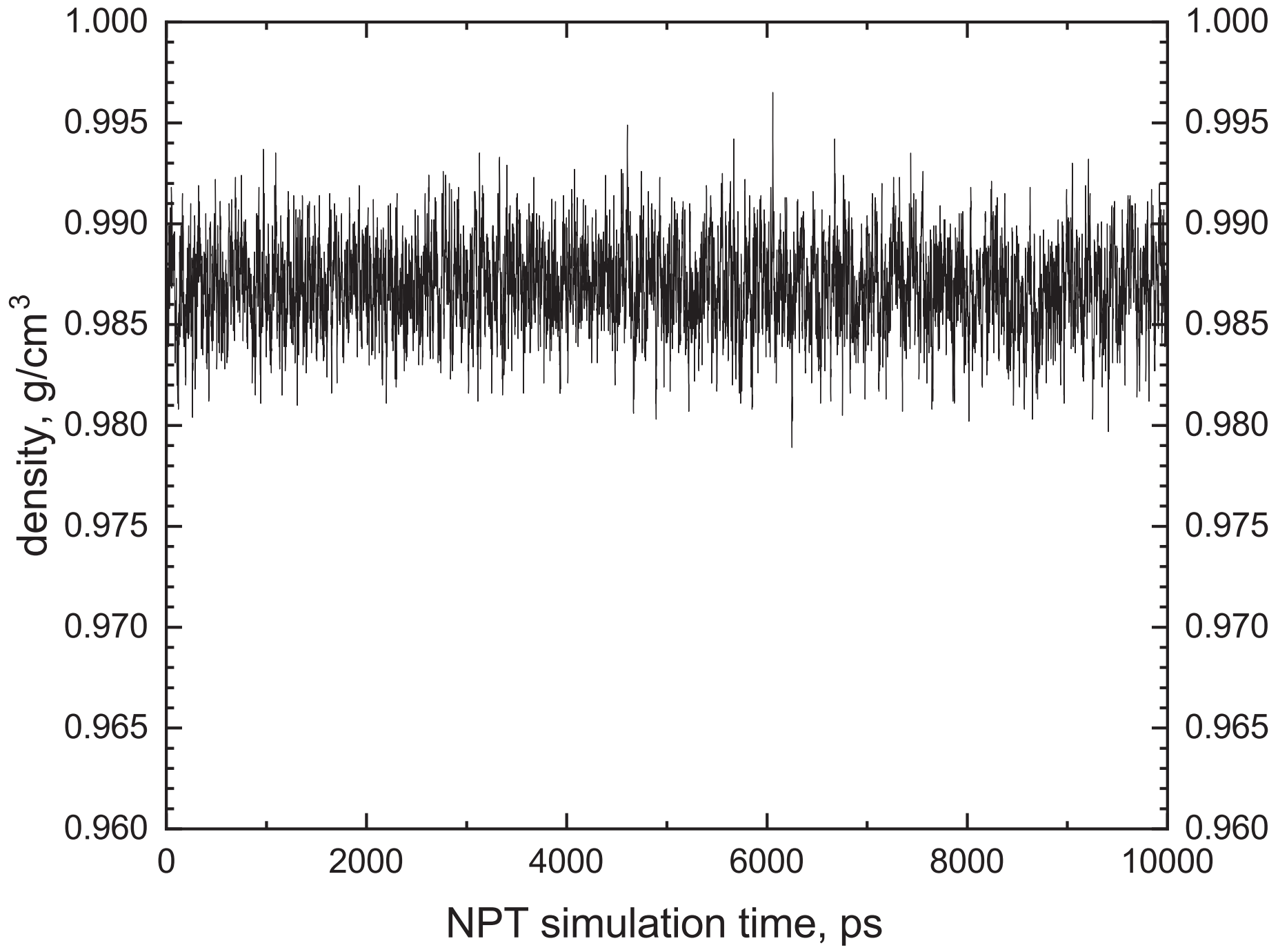


Figure S1. Evolution of the density in Run1 simulation for aqueous solution of *trans* proline isomer of the Ala-Glu-Pro-Phe peptide.

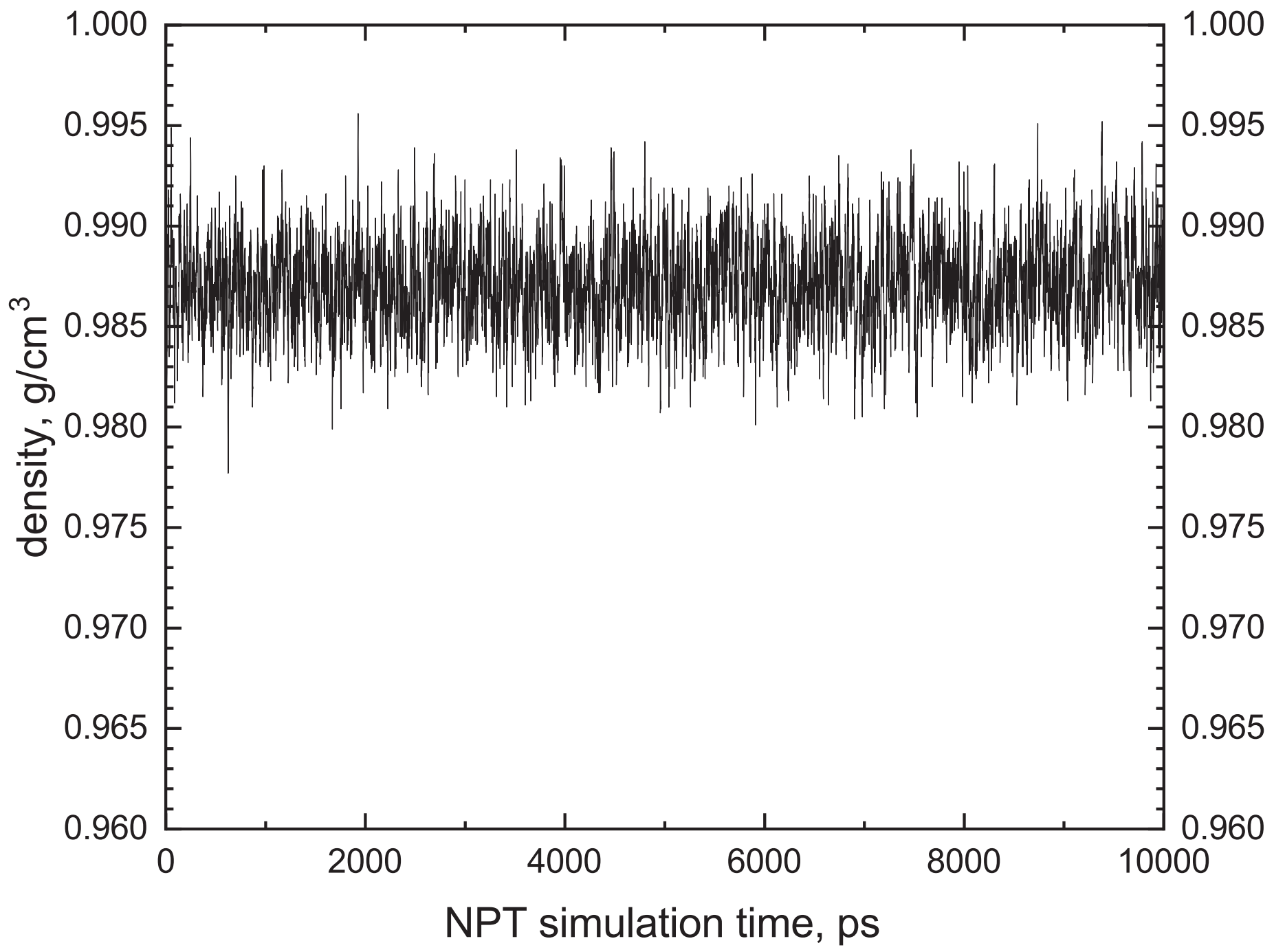


Figure S2. Evolution of the density in Run1 simulation for aqueous solution of *cis* proline isomer of the Ala-Glu-Pro-Phe peptide.

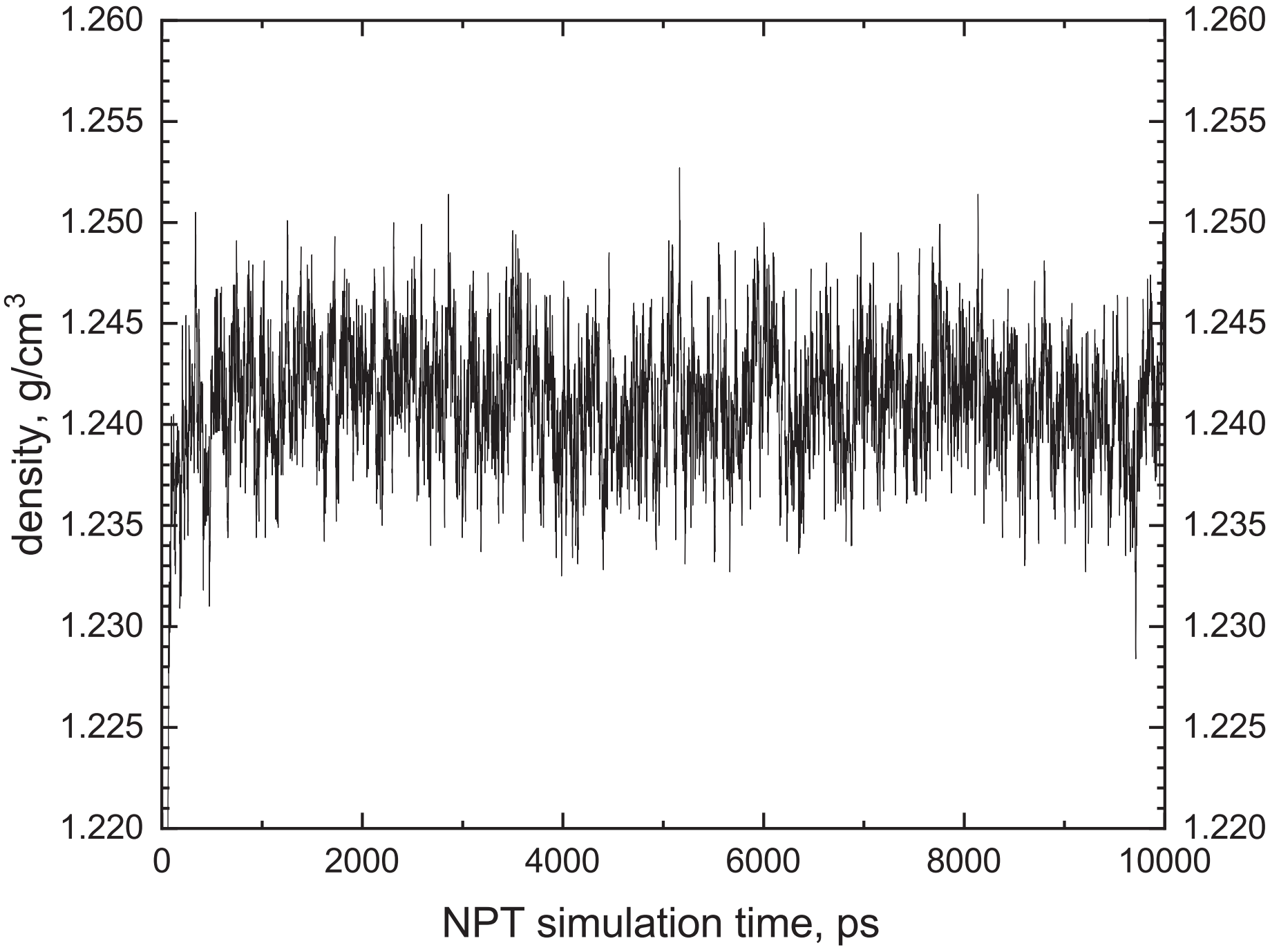


Figure S3. Evolution of the density in Run1 simulation for aqueous [EMim][TFA] mixture of *trans* proline isomer of the Ala-Glu-Pro-Phe peptide.

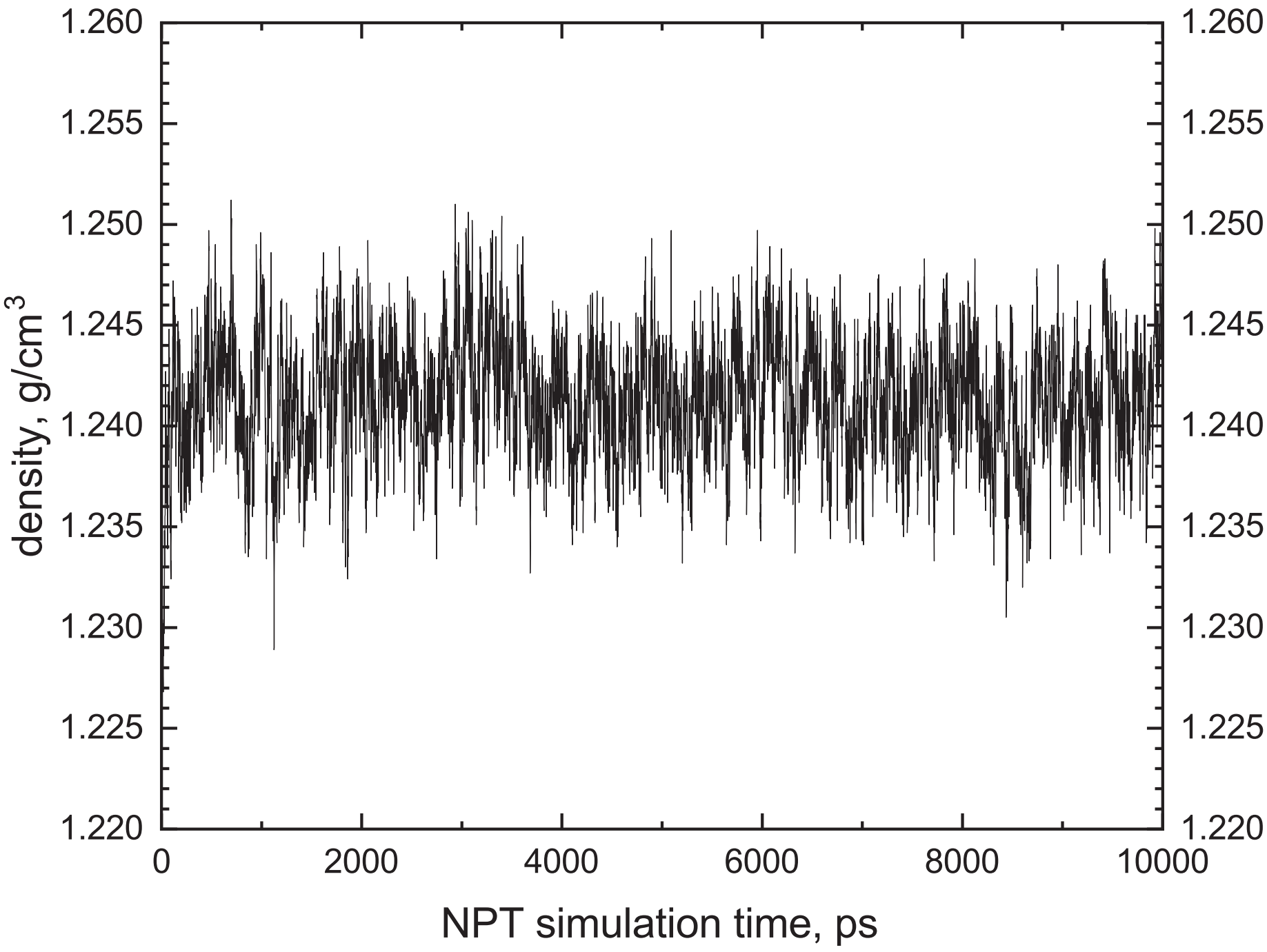


Figure S4. Evolution of the density in Run2 simulation for aqueous [EMim][TFA] mixture of *trans* proline isomer of the Ala-Glu-Pro-Phe peptide.

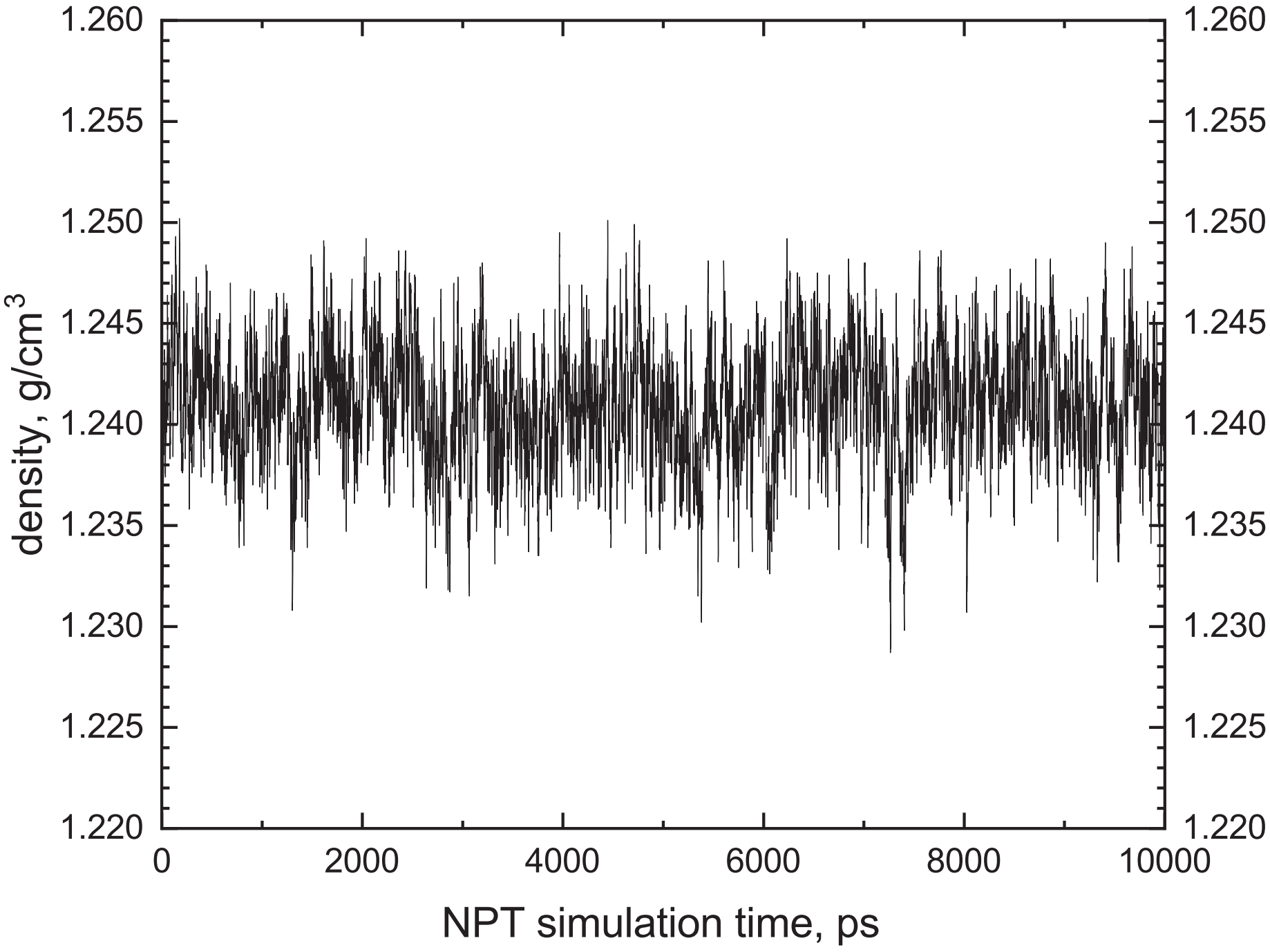


Figure S5. Evolution of the density in Run3 simulation for aqueous [EMim][TFA] mixture of *trans* proline isomer of the Ala-Glu-Pro-Phe peptide.

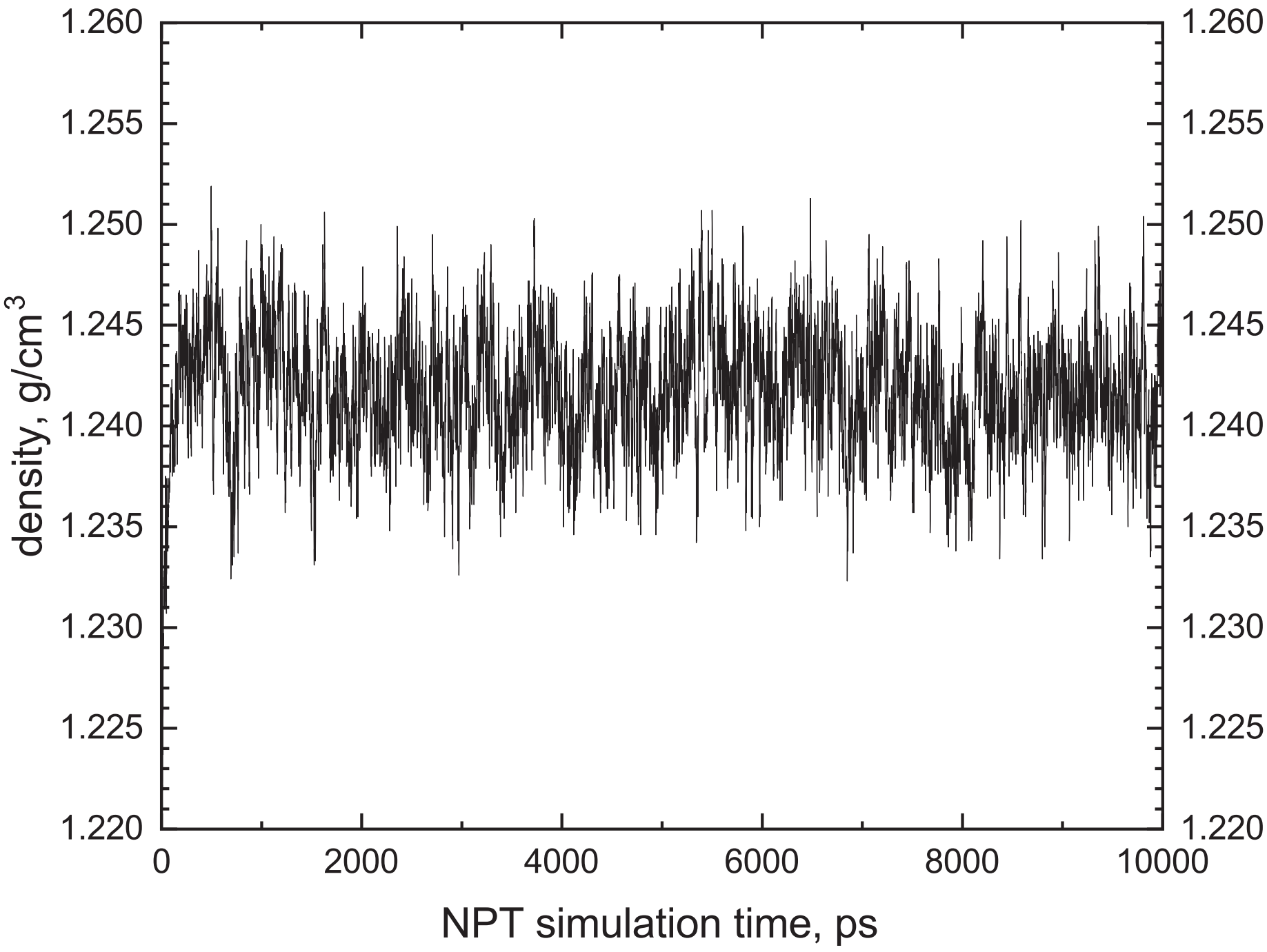


Figure S6. Evolution of the density in Run4 simulation for aqueous [EMim][TFA] mixture of *trans* proline isomer of the Ala-Glu-Pro-Phe peptide.

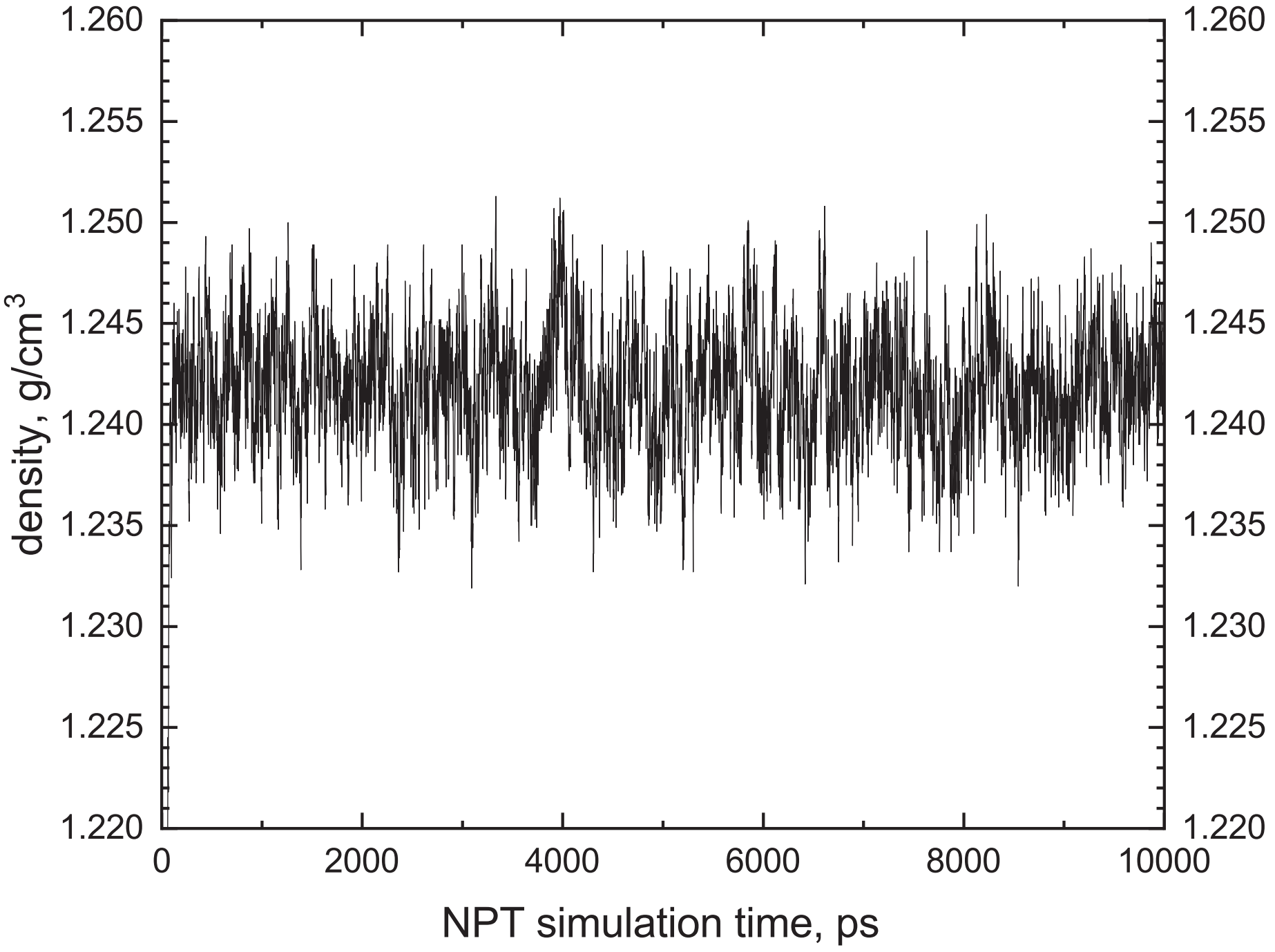


Figure S7. Evolution of the density in Run1 simulation for aqueous [EMim][TFA] mixture of *cis* proline isomer of the Ala-Glu-Pro-Phe peptide.

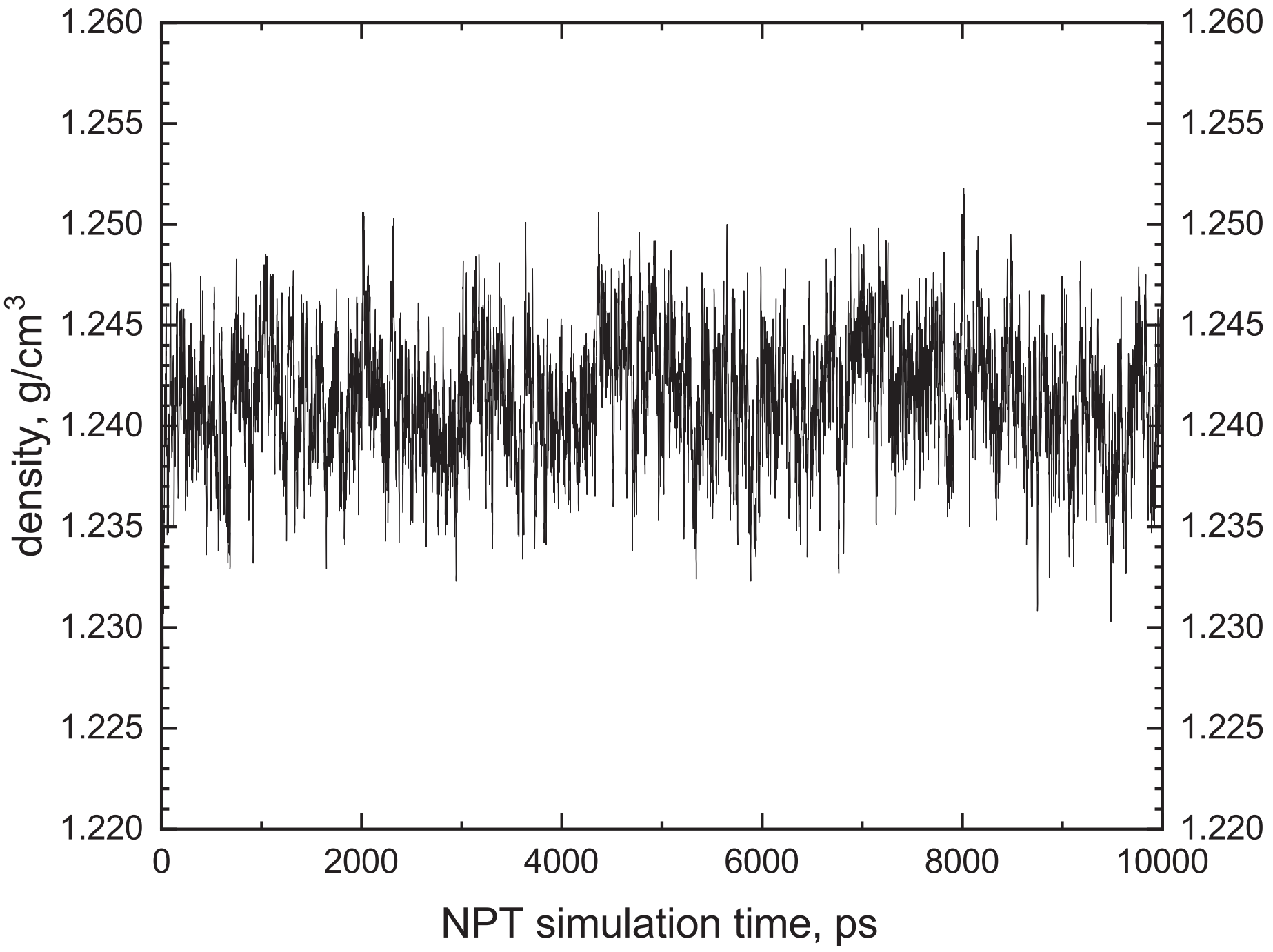


Figure S8. Evolution of the density in Run2 simulation for aqueous [EMim][TFA] mixture of *cis* proline isomer of the Ala-Glu-Pro-Phe peptide.

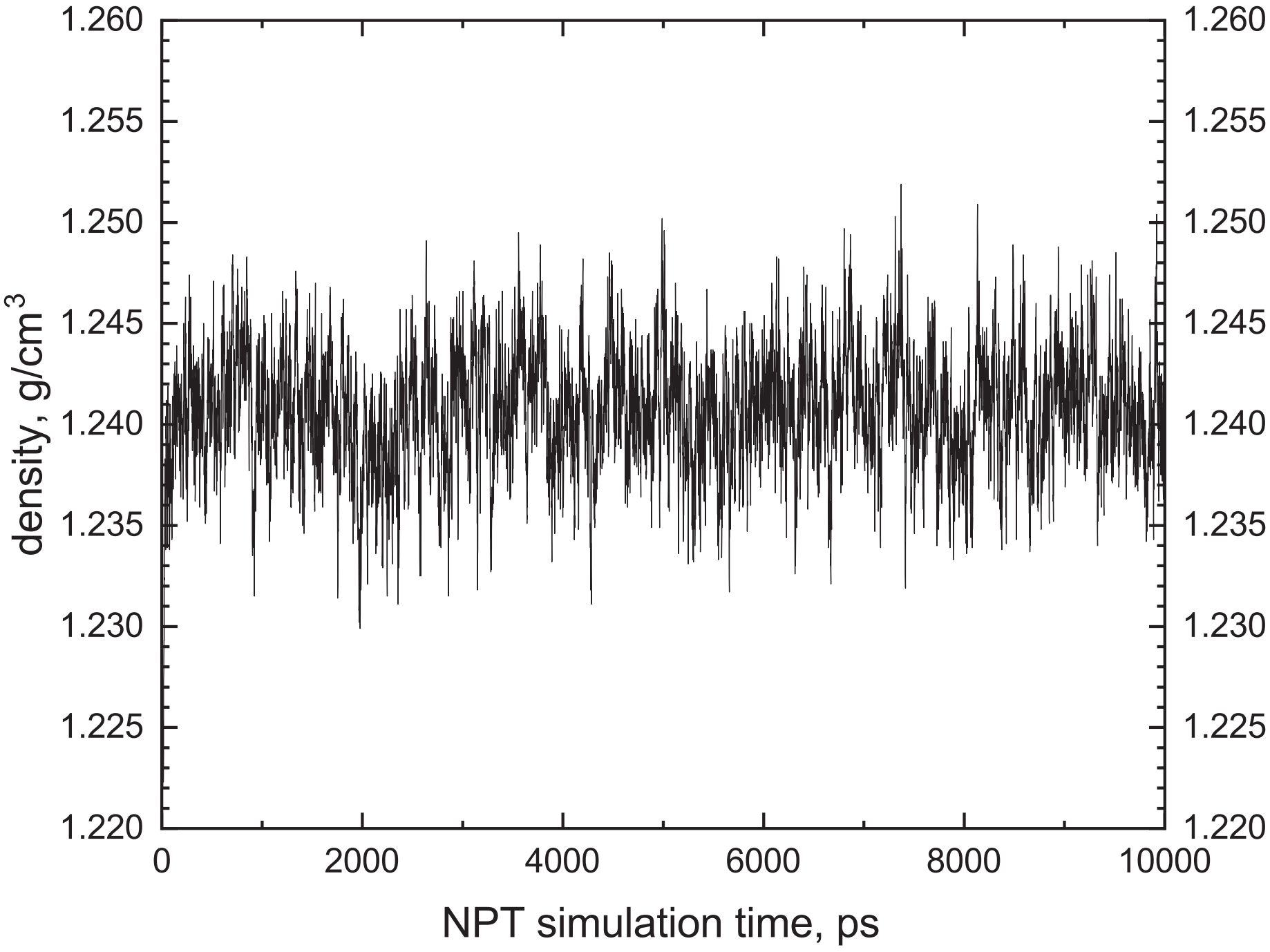


Figure S9. Evolution of the density in Run3 simulation for aqueous [EMim][TFA] mixture of *cis* proline isomer of the Ala-Glu-Pro-Phe peptide.

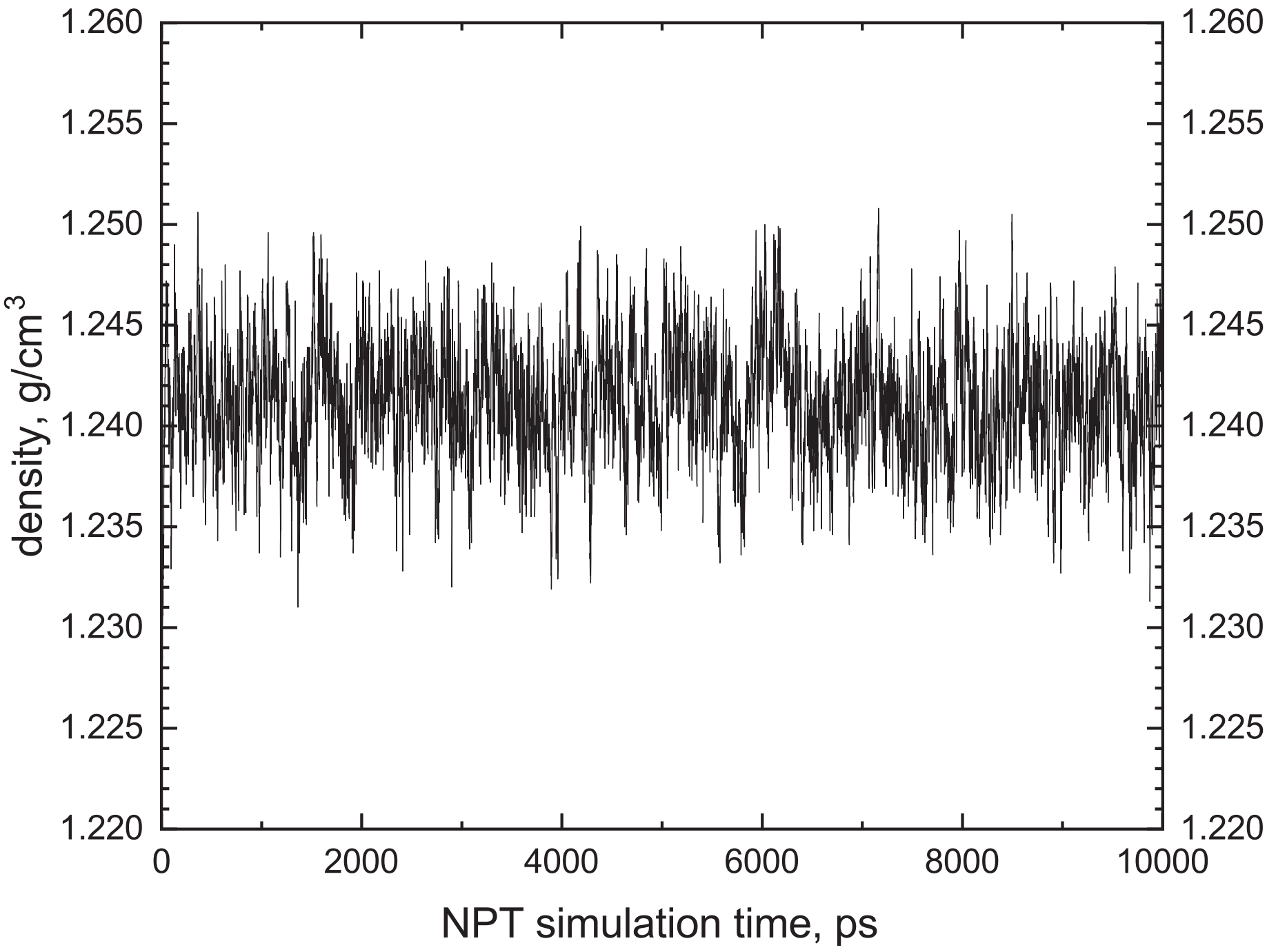


Figure S10. Evolution of the density in Run4 simulation for aqueous [EMim][TFA] mixture of *cis* proline isomer of the Ala-Glu-Pro-Phe peptide.

# 2. Evolution of the Ramachandran angles

## 2.1 *Trans*- and *cis*-AEPF in aqueous solution

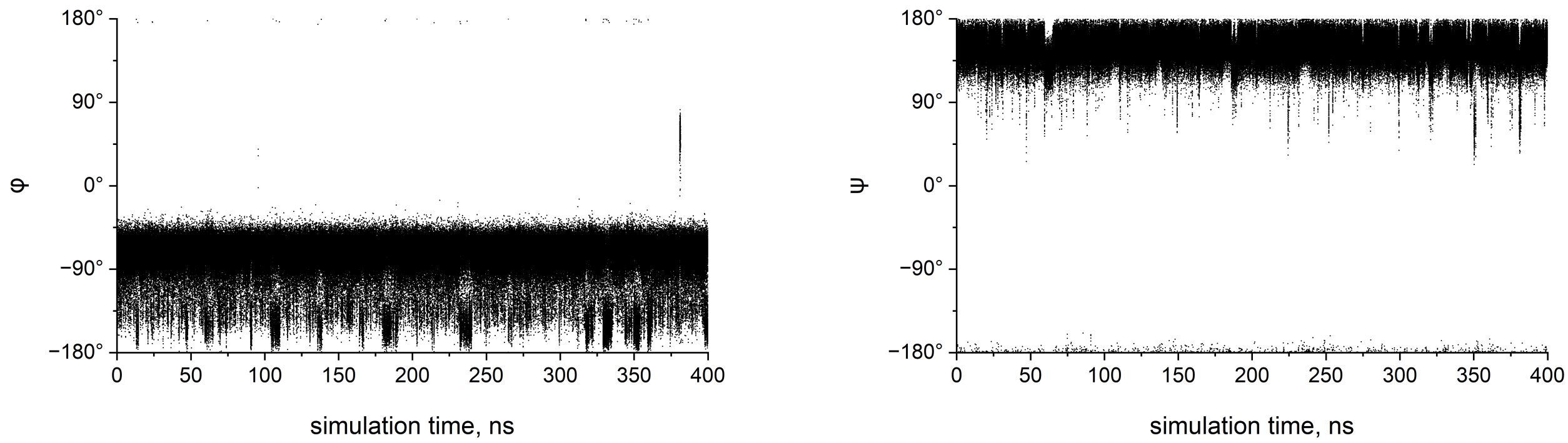


(a) (b)

Figure S11. Evolution of the Ramachandran angles $\varphi$ (a) and $\psi$ (b) of the Glu residue during the production stage of Run1 simulation for aqueous solution of *trans* proline isomer of the Ala-Glu-Pro-Phe peptide.

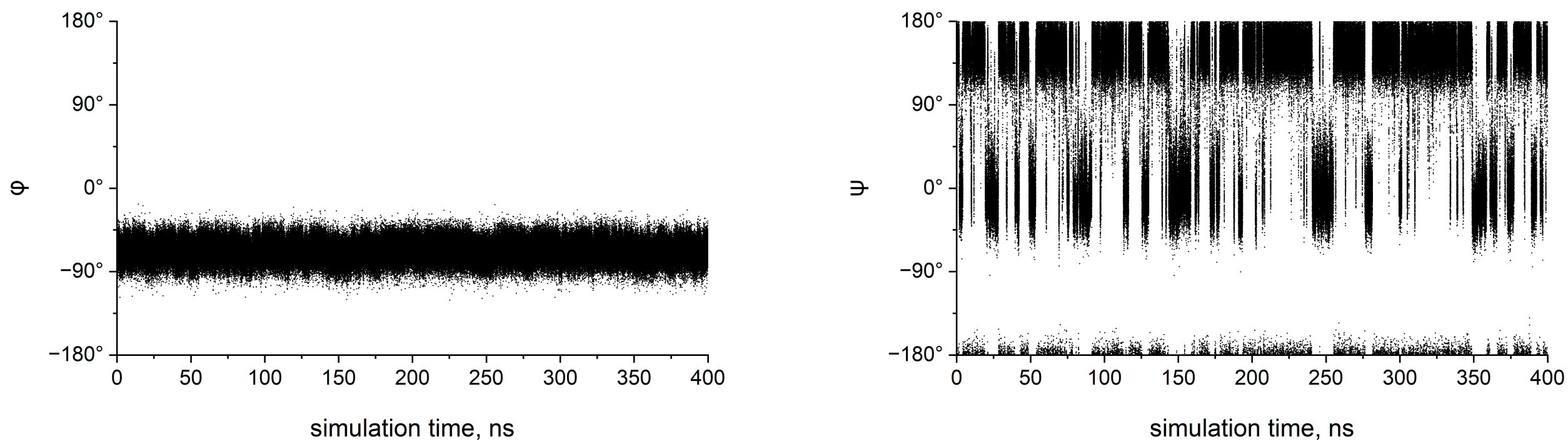


(a) (b)

Figure S12. Evolution of the Ramachandran angles $\varphi$ (a) and $\psi$ (b) of the Pro residue during the production stage of Run1 simulation for aqueous solution of *trans* proline isomer of the Ala-Glu-Pro-Phe peptide.

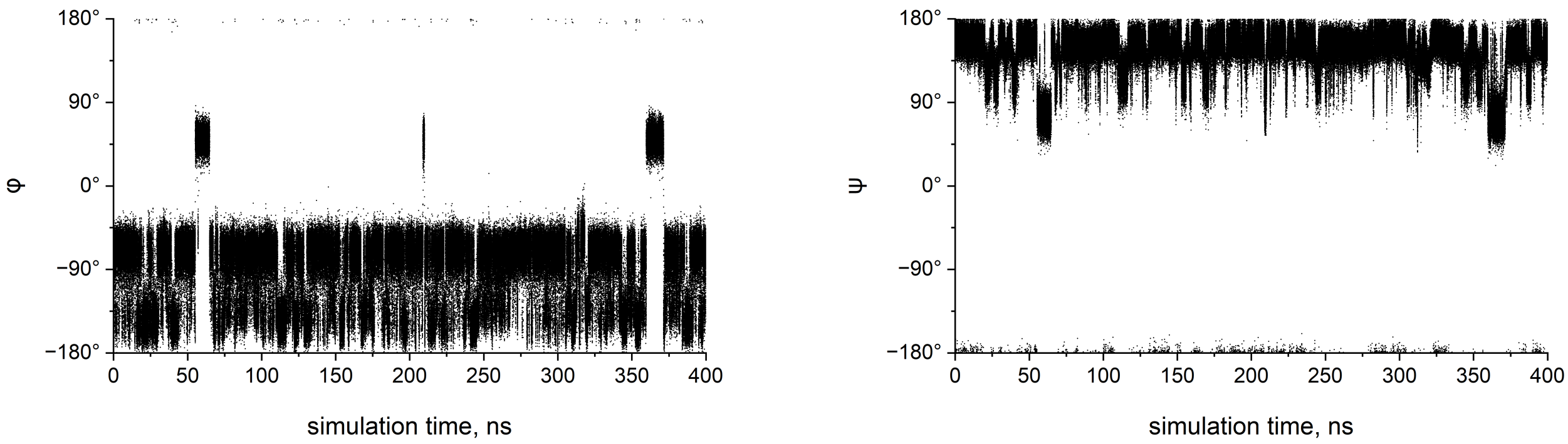


(a) (b)

Figure S13. Evolution of the Ramachandran angles $\varphi$ (a) and $\psi$ (b) of the Glu residue during the production stage of Run1 simulation for aqueous solution of *cis* proline isomer of the Ala-Glu-Pro-Phe peptide.

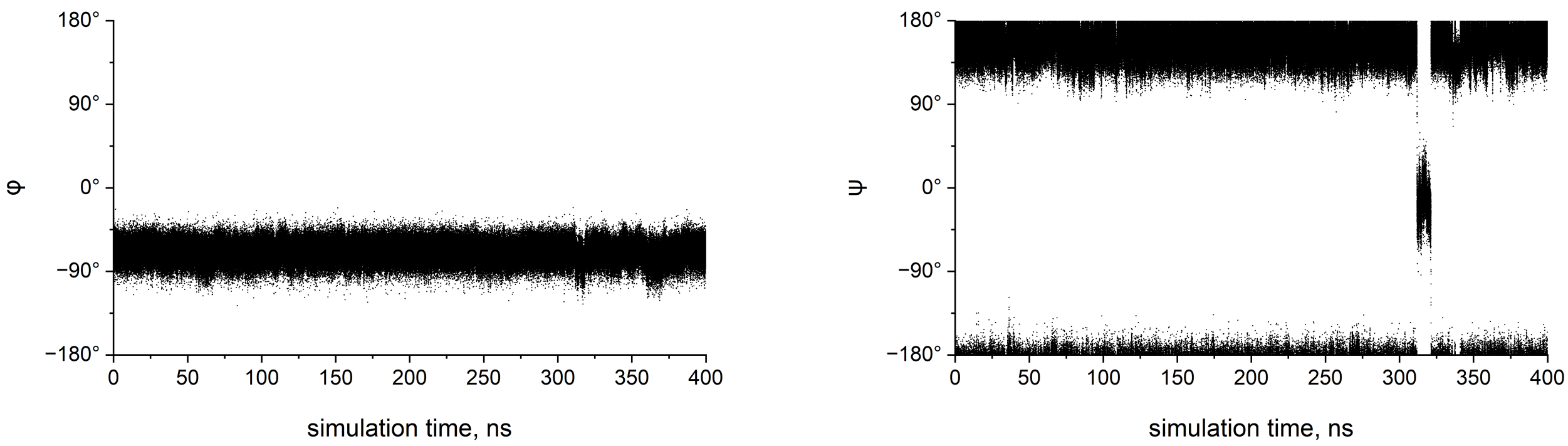


(a) (b)

Figure S14. Evolution of the Ramachandran angles $\varphi$ (a) and $\psi$ (b) of the Pro residue during the production stage of Run1 simulation for aqueous solution of *cis* proline isomer of the Ala-Glu-Pro-Phe peptide.

## 2.2 *Trans*-AEPF in aqueous ionic liquid solution

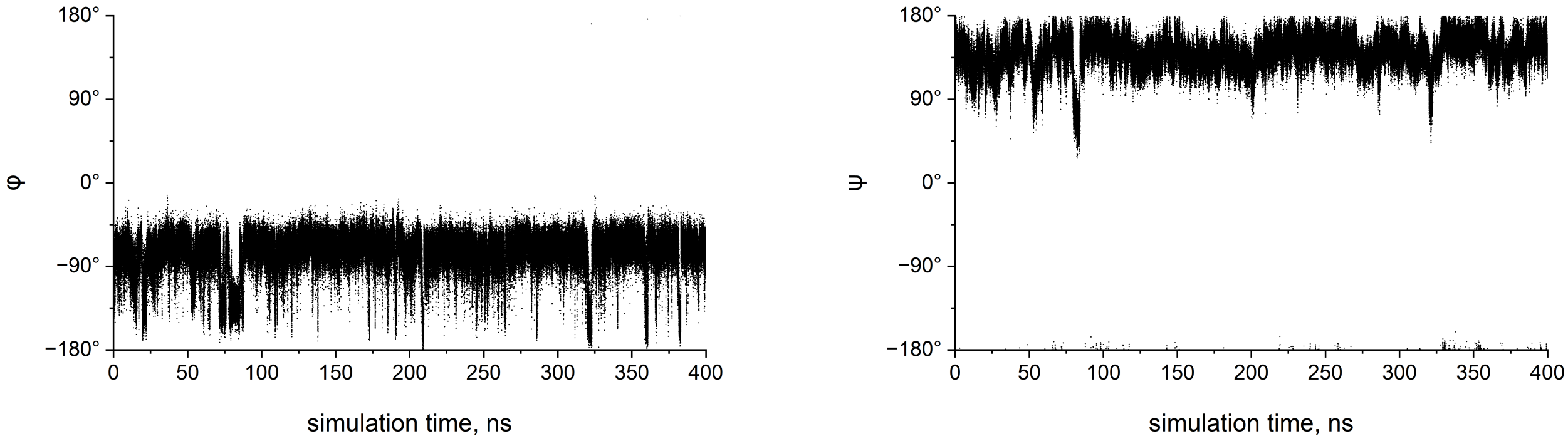


(a) (b)

Figure S15. Evolution of the Ramachandran angles $\varphi$ (a) and $\psi$ (b) of the Glu residue during the production stage of Run1 simulation for aqueous [EMim][TFA] mixture of *trans* proline isomer of the Ala-Glu-Pro-Phe peptide.

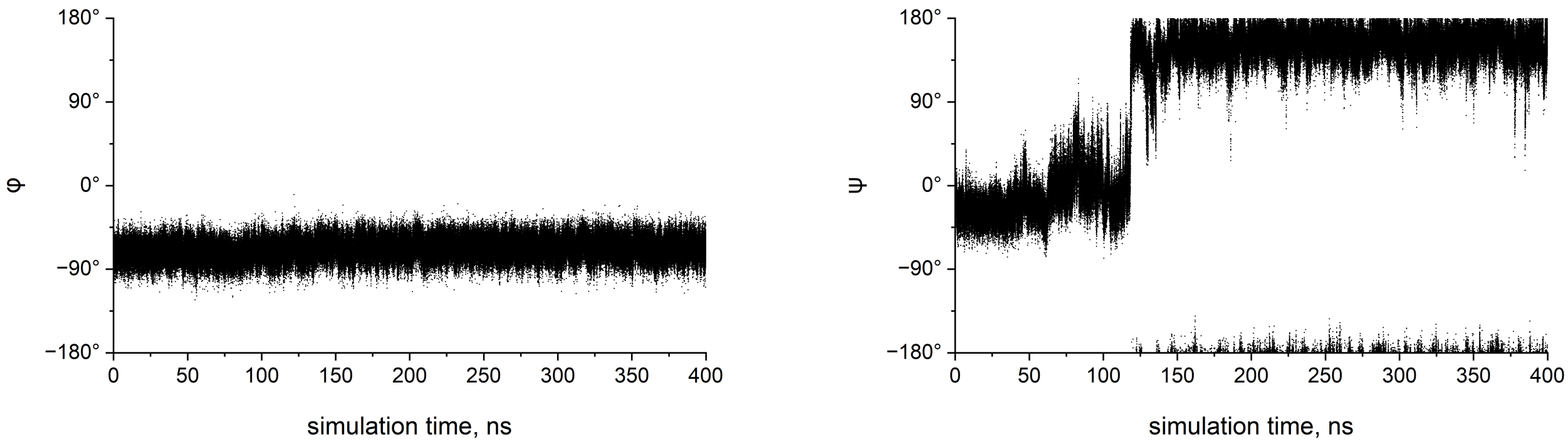


(a) (b)

Figure S16. Evolution of the Ramachandran angles $\varphi$ (a) and $\psi$ (b) of the Pro residue during the production stage of Run1 simulation for aqueous [EMim][TFA] mixture of *trans* proline isomer of the Ala-Glu-Pro-Phe peptide.

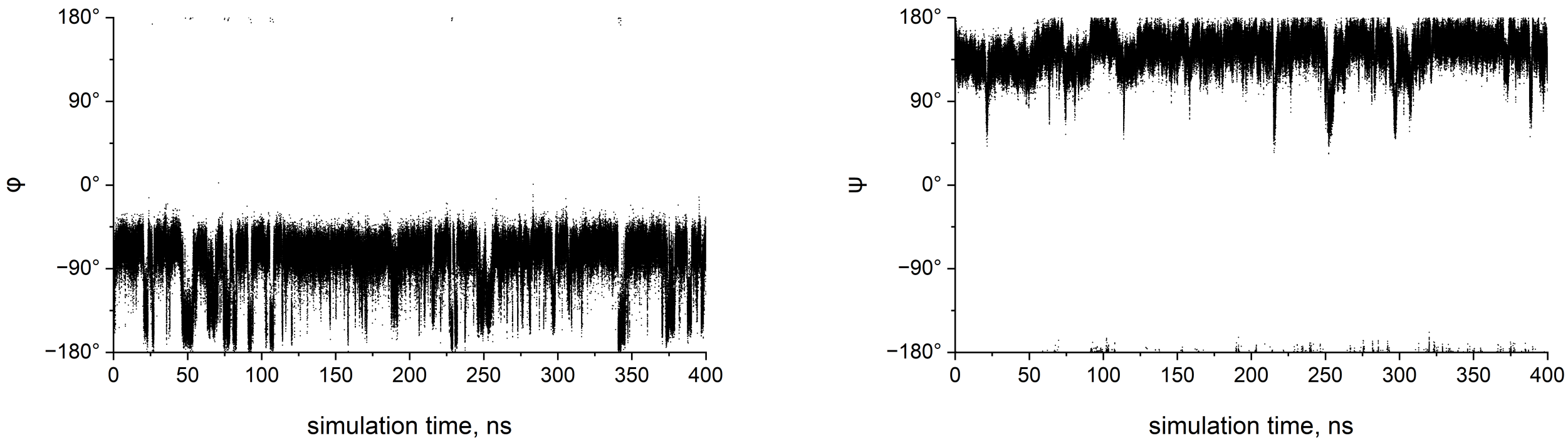


(a) (b)

Figure S17. Evolution of the Ramachandran angles $\varphi$ (a) and $\psi$ (b) of the Glu residue during the production stage of Run2 simulation for aqueous [EMim][TFA] mixture of *trans* proline isomer of the Ala-Glu-Pro-Phe peptide.

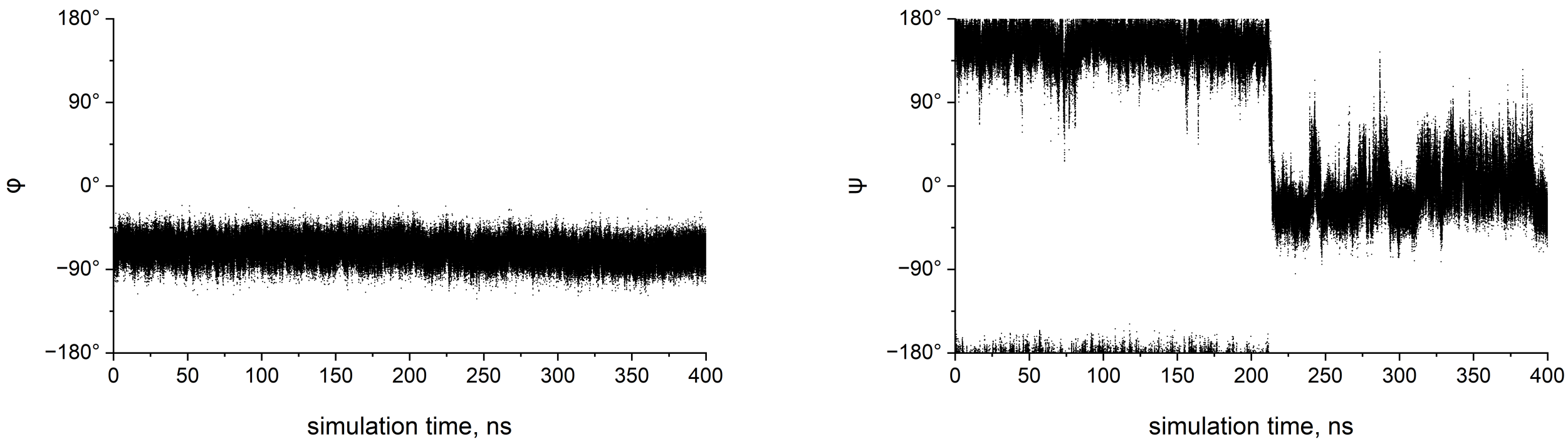


(a) (b)

Figure S18. Evolution of the Ramachandran angles $\varphi$ (a) and $\psi$ (b) of the Pro residue during the production stage of Run2 simulation for aqueous [EMim][TFA] mixture of *trans* proline isomer of the Ala-Glu-Pro-Phe peptide.

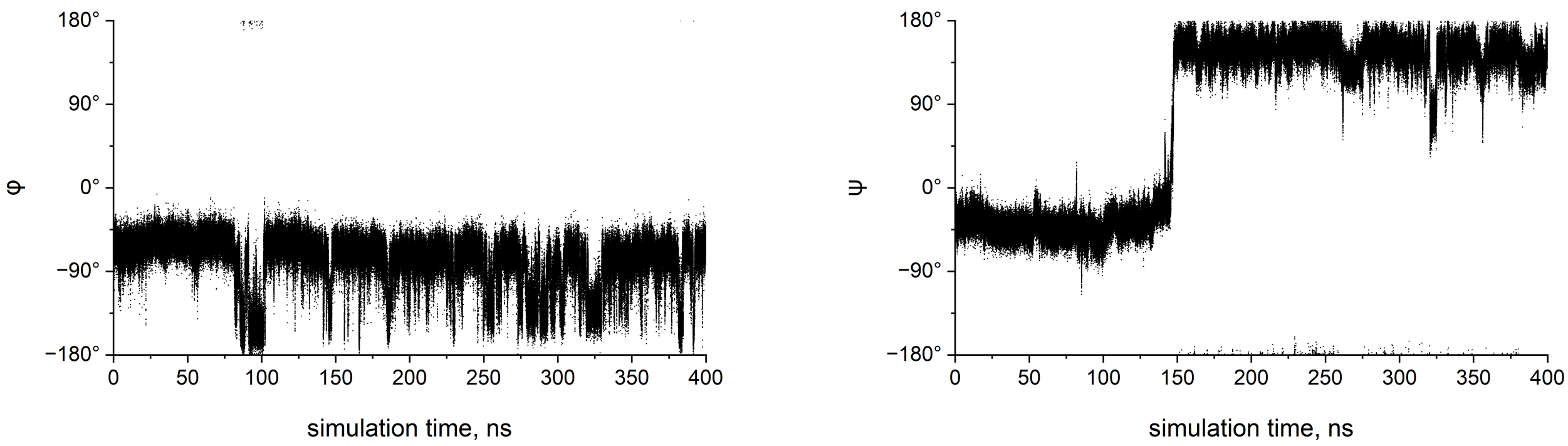


(a) (b)

Figure S19. Evolution of the Ramachandran angles $\varphi$ (a) and $\psi$ (b) of the Glu residue during the production stage of Run3 simulation for aqueous [EMim][TFA] mixture of *trans* proline isomer of the Ala-Glu-Pro-Phe peptide.

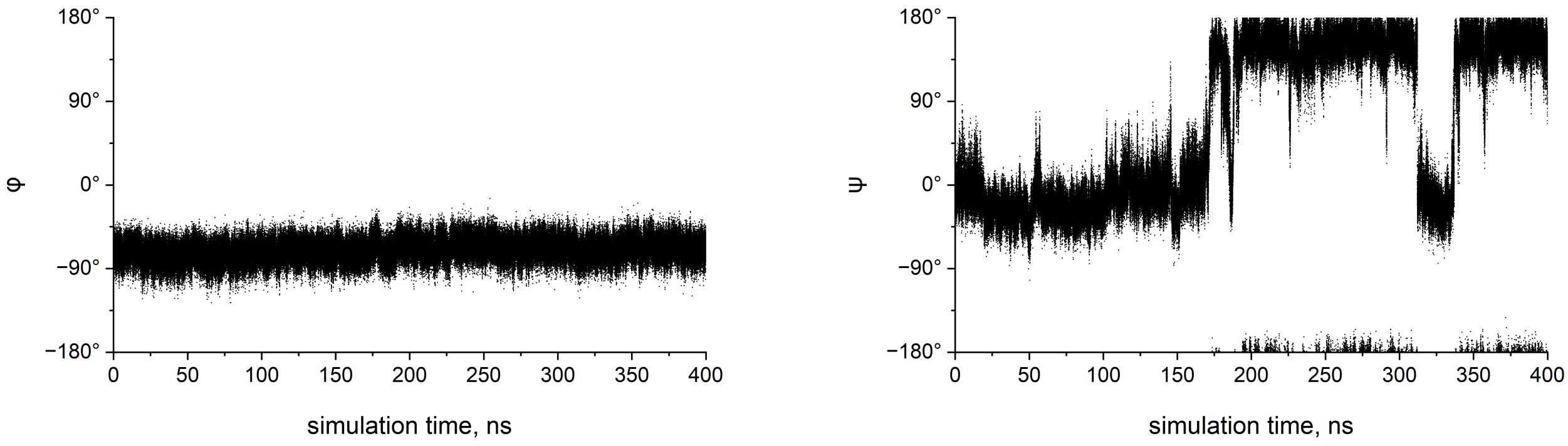


(a) (b)

Figure S20. Evolution of the Ramachandran angles $\varphi$ (a) and $\psi$ (b) of the Pro residue during the production stage of Run3 simulation for aqueous [EMim][TFA] mixture of *trans* proline isomer of the Ala-Glu-Pro-Phe peptide.

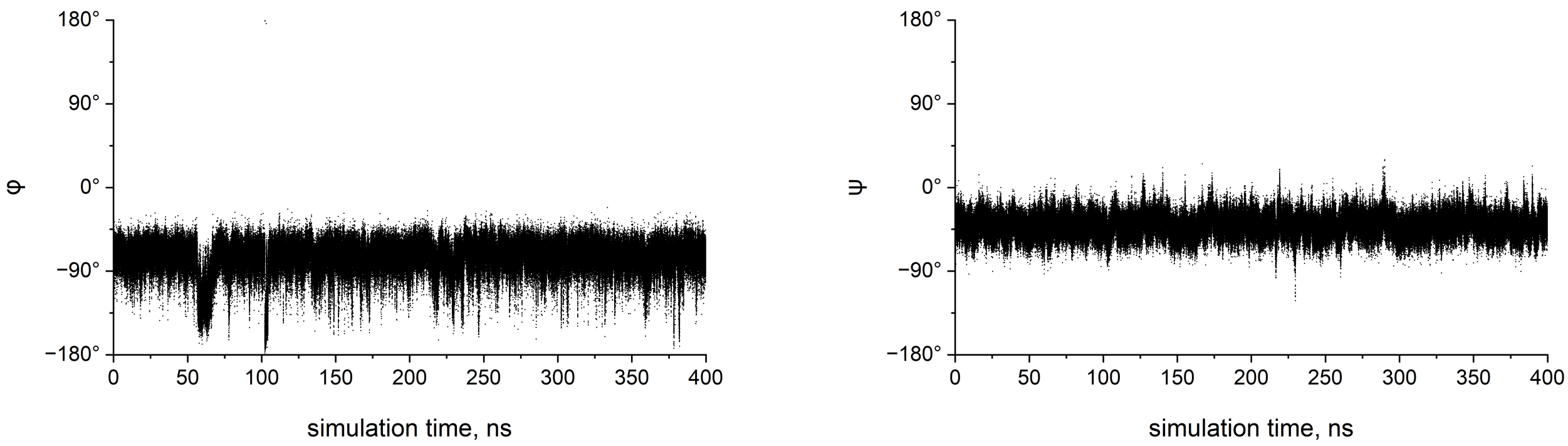


(a) (b)

Figure S21. Evolution of the Ramachandran angles $\varphi$ (a) and $\psi$ (b) of the Glu residue during the production stage of Run4 simulation for aqueous [EMim][TFA] mixture of *trans* proline isomer of the Ala-Glu-Pro-Phe peptide.

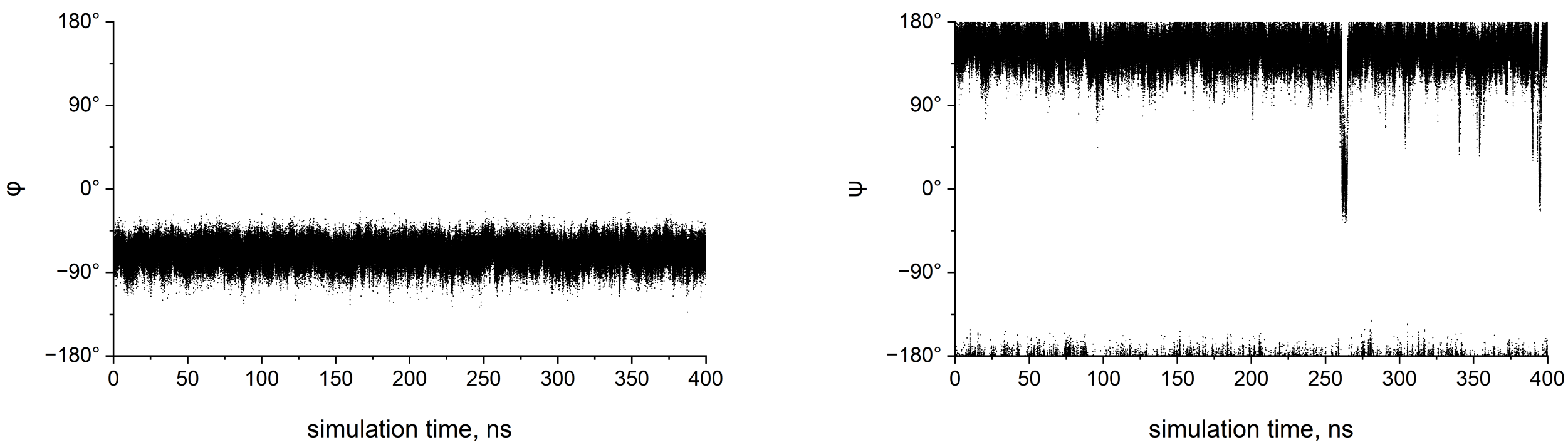


(a) (b)

Figure S22. Evolution of the Ramachandran angles $\varphi$ (a) and $\psi$ (b) of the Pro residue during the production stage of Run4 simulation for aqueous [EMim][TFA] mixture of *trans* proline isomer of the Ala-Glu-Pro-Phe peptide.

## 2.3 *Cis*-AEPF in aqueous ionic liquid solution

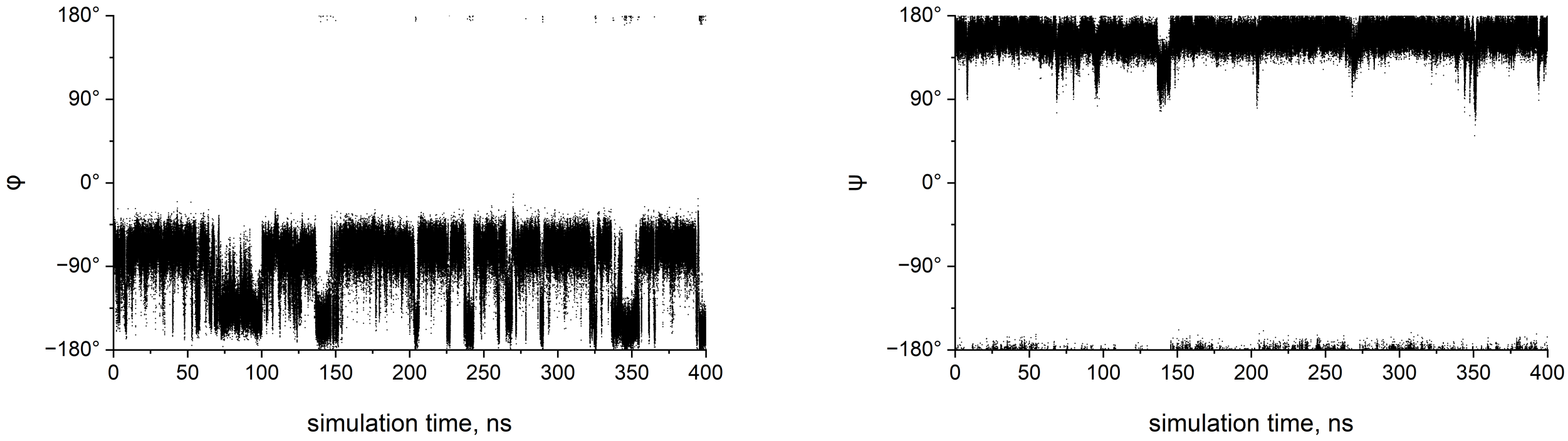


(a) (b)

Figure S23. Evolution of the Ramachandran angles $\varphi$ (a) and $\psi$ (b) of the Glu residue during the production stage of Run1 simulation for aqueous [EMim][TFA] mixture of *cis* proline isomer of the Ala-Glu-Pro-Phe peptide.

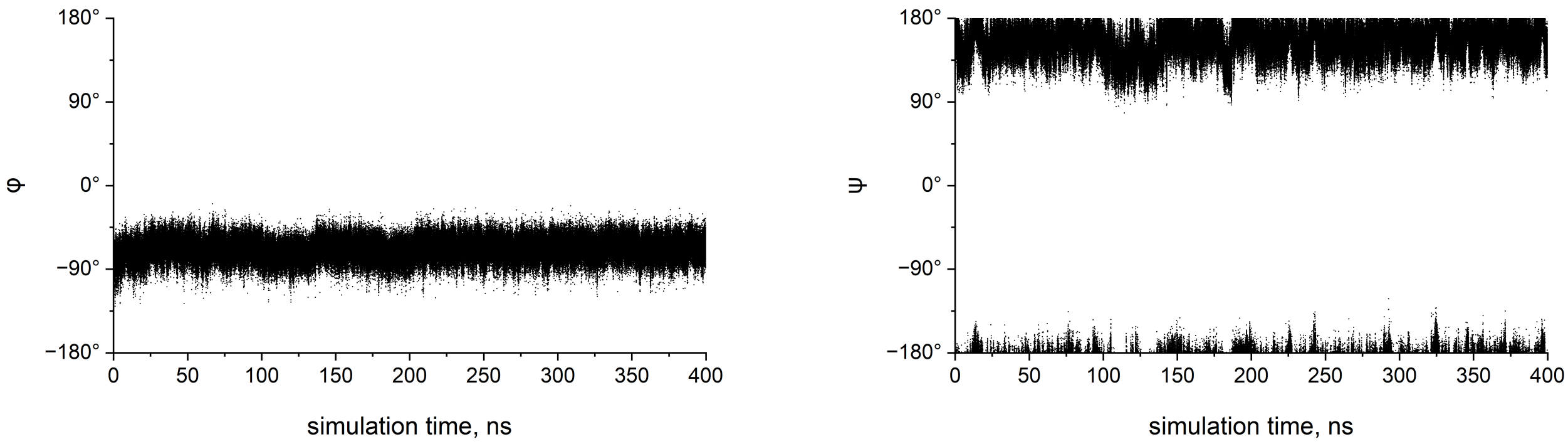


(a) (b)

Figure S24. Evolution of the Ramachandran angles $\varphi$ (a) and $\psi$ (b) of the Pro residue during the production stage of Run1 simulation for aqueous [EMim][TFA] mixture of *cis* proline isomer of the Ala-Glu-Pro-Phe peptide.

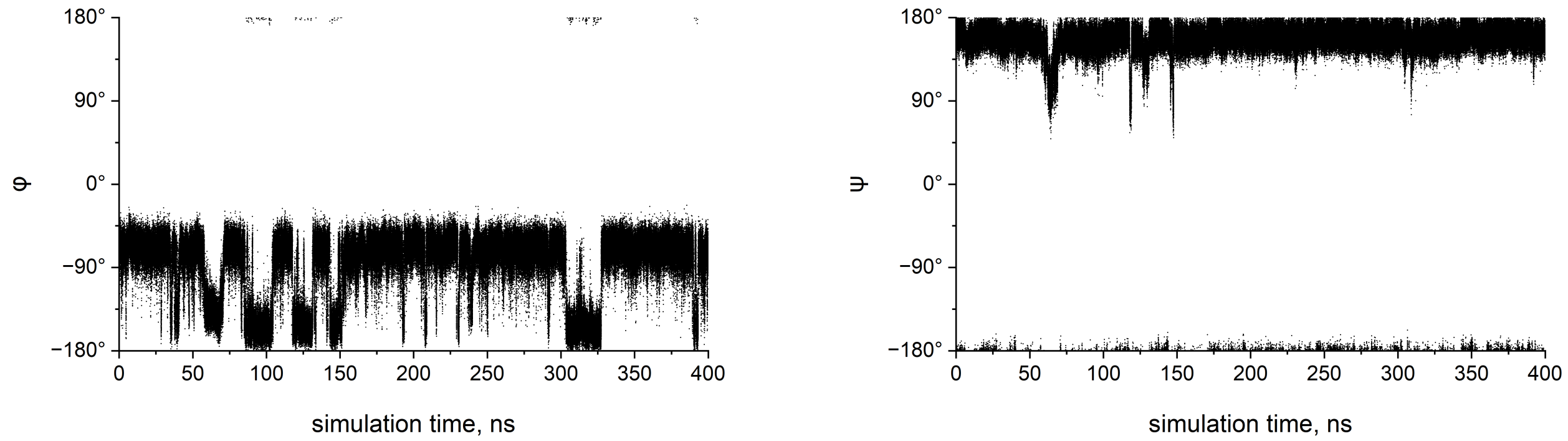


(a) (b)

Figure S25. Evolution of the Ramachandran angles $\varphi$ (a) and $\psi$ (b) of the Glu residue during the production stage of Run2 simulation for aqueous [EMim][TFA] mixture of *cis* proline isomer of the Ala-Glu-Pro-Phe peptide.

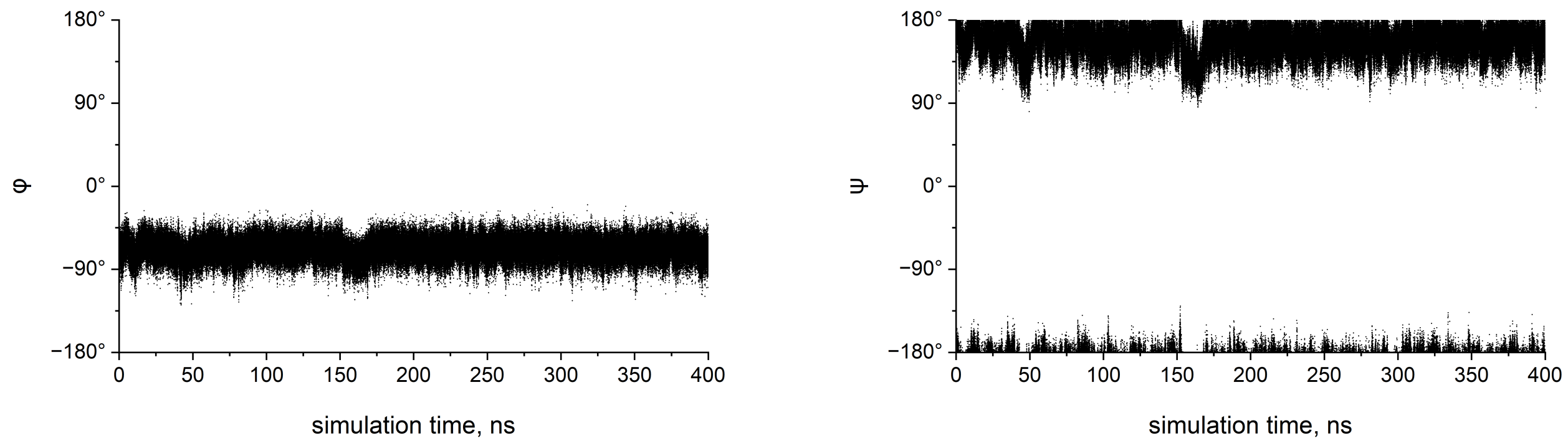


(a) (b)

Figure S26. Evolution of the Ramachandran angles $\varphi$ (a) and $\psi$ (b) of the Pro residue during the production stage of Run2 simulation for aqueous [EMim][TFA] mixture of *cis* proline isomer of the Ala-Glu-Pro-Phe peptide.

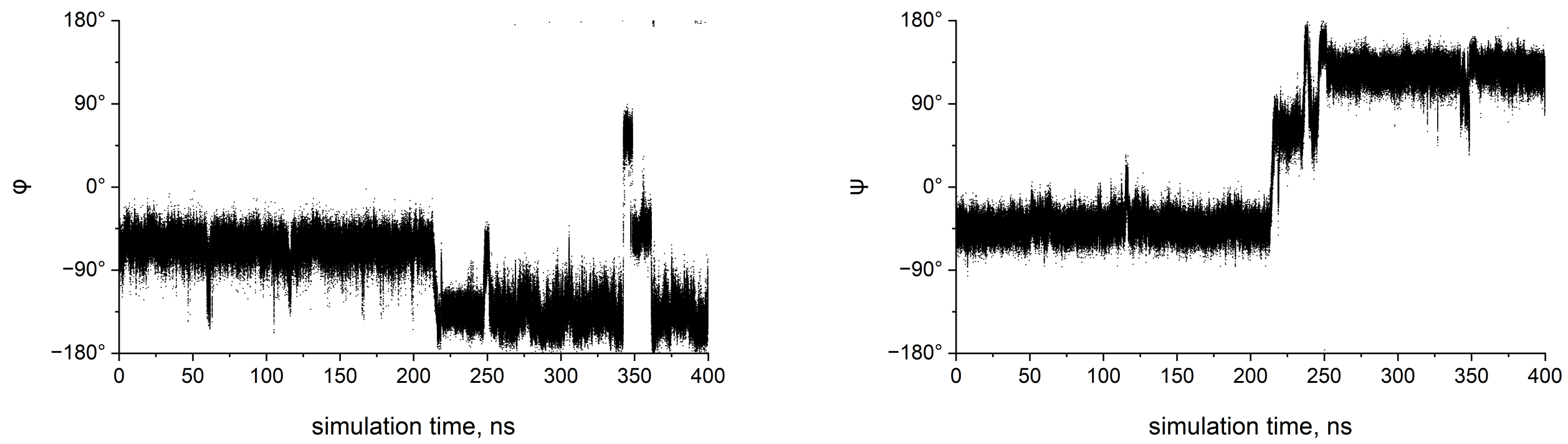


(a) (b)

Figure S27. Evolution of the Ramachandran angles $\varphi$ (a) and $\psi$ (b) of the Glu residue during the production stage of Run3 simulation for aqueous [EMim][TFA] mixture of *cis* proline isomer of the Ala-Glu-Pro-Phe peptide.

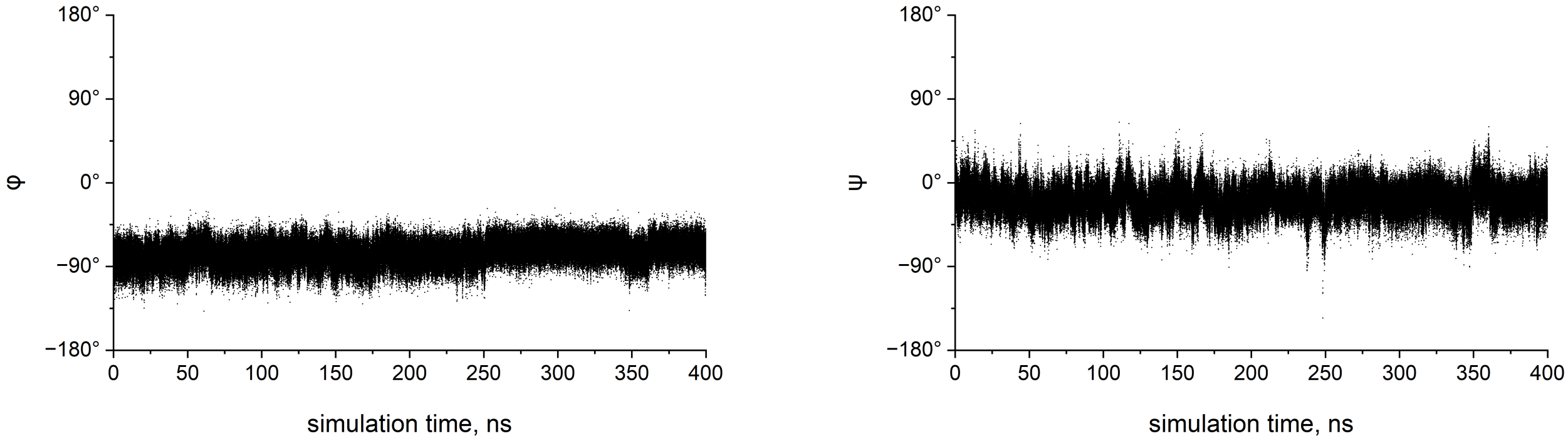


(a) (b)

Figure S28. Evolution of the Ramachandran angles $\varphi$ (a) and $\psi$ (b) of the Pro residue during the production stage of Run3 simulation for aqueous [EMim][TFA] mixture of *cis* proline isomer of the Ala-Glu-Pro-Phe peptide.

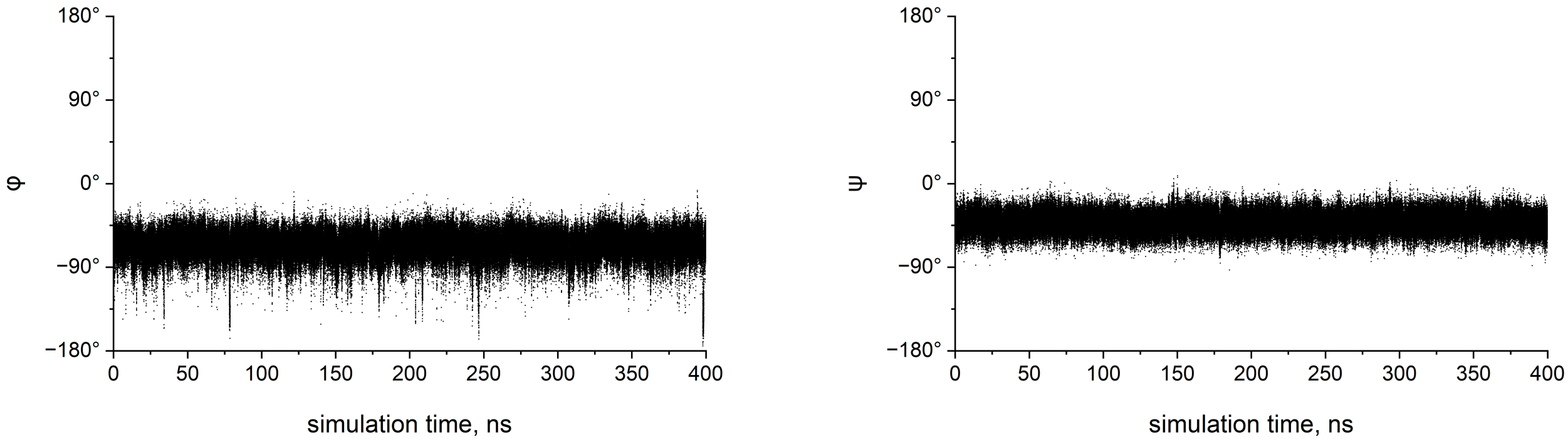


(a) (b)

Figure S29. Evolution of the Ramachandran angles $\varphi$ (a) and $\psi$ (b) of the Glu residue during the production stage of Run4 simulation for aqueous [EMim][TFA] mixture of *cis* proline isomer of the Ala-Glu-Pro-Phe peptide.

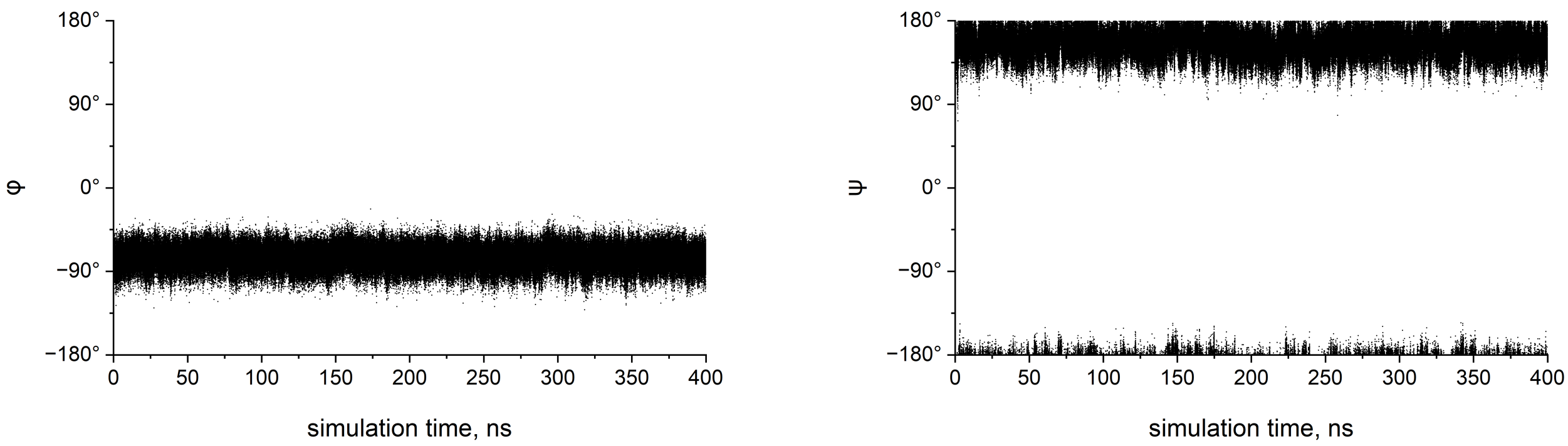


(a) (b)

Figure S30. Evolution of the Ramachandran angles $\varphi$ (a) and $\psi$ (b) of the Pro residue during the production stage of Run4 simulation for aqueous [EMim][TFA] mixture of *cis* proline isomer of the Ala-Glu-Pro-Phe peptide.

## 3. Details of the QM/MM calculations

Table S1. Statistical information concerning the amounts and types of solvent molecules included to the QM region in the QM/MM calculations of the $^{1}$H NMR isotropic shielding constants for *trans*- and *cis*-AEPF peptides. The minimum, maximum and median values of the numbers of molecules of each type have been provided. Each series of QM/MM calculations was performed for a set of 100 molecular snapshots. Separate series of QM/MM calculations were performed for four distinct conformations of the AEPF peptide in aqueous IL solution, as indicated in the second column. A single series of QM/MM calculations for each isomer of AEPF in aqueous solution were performed, refer to the main text of the article for details.

| | | Aqueous solution | | | Aqueous [EMim][TFA] solution | | | | | | | | |
|---|---|---|---|---|---|---|---|---|---|---|---|---|---|
| | | $H_2O$ | | | $H_2O$ | | | $EMim^+$ | | | $TFA^-$ | | |
| | | min | max | median | min | max | median | min | max | median | min | max | median |
| *trans*-AEPF | Glu $P_{II}$, Pro $P_{II}$ | 42 | 64 | 54 | 12 | 25 | 19 | 5 | 12 | 9 | 1 | 8 | 4.5 |
| | Glu $P_{II}$, Pro $\delta$ | | | | 10 | 24 | 17 | 6 | 11 | 8 | 1 | 8 | 4.5 |
| | Glu $\delta$, Pro $P_{II}$ | | | | 11 | 26 | 18 | 5 | 12 | 9 | 1 | 9 | 4 |
| | Glu $\delta$, Pro $\delta$ | | | | 9 | 24 | 16 | 5 | 11 | 8 | 2 | 7 | 4 |
| *cis*-AEPF | Glu $P_{II}$, Pro $P_{II}$ | 40 | 62 | 52 | 10 | 23 | 18 | 5 | 13 | 9 | 1 | 7 | 4 |
| | Glu $\beta$, Pro $\delta$ | | | | 8 | 23 | 15 | 3 | 12 | 9 | 1 | 8 | 4 |
| | Glu $\delta$, Pro $P_{II}$ | | | | 12 | 24 | 18 | 5 | 12 | 8 | 1 | 7 | 4 |
| | Glu $\delta$, Pro $\delta$ | | | | 10 | 23 | 18 | 5 | 13 | 9 | 1 | 7 | 4 |

# 4. The pattern of specific intermolecular interactions

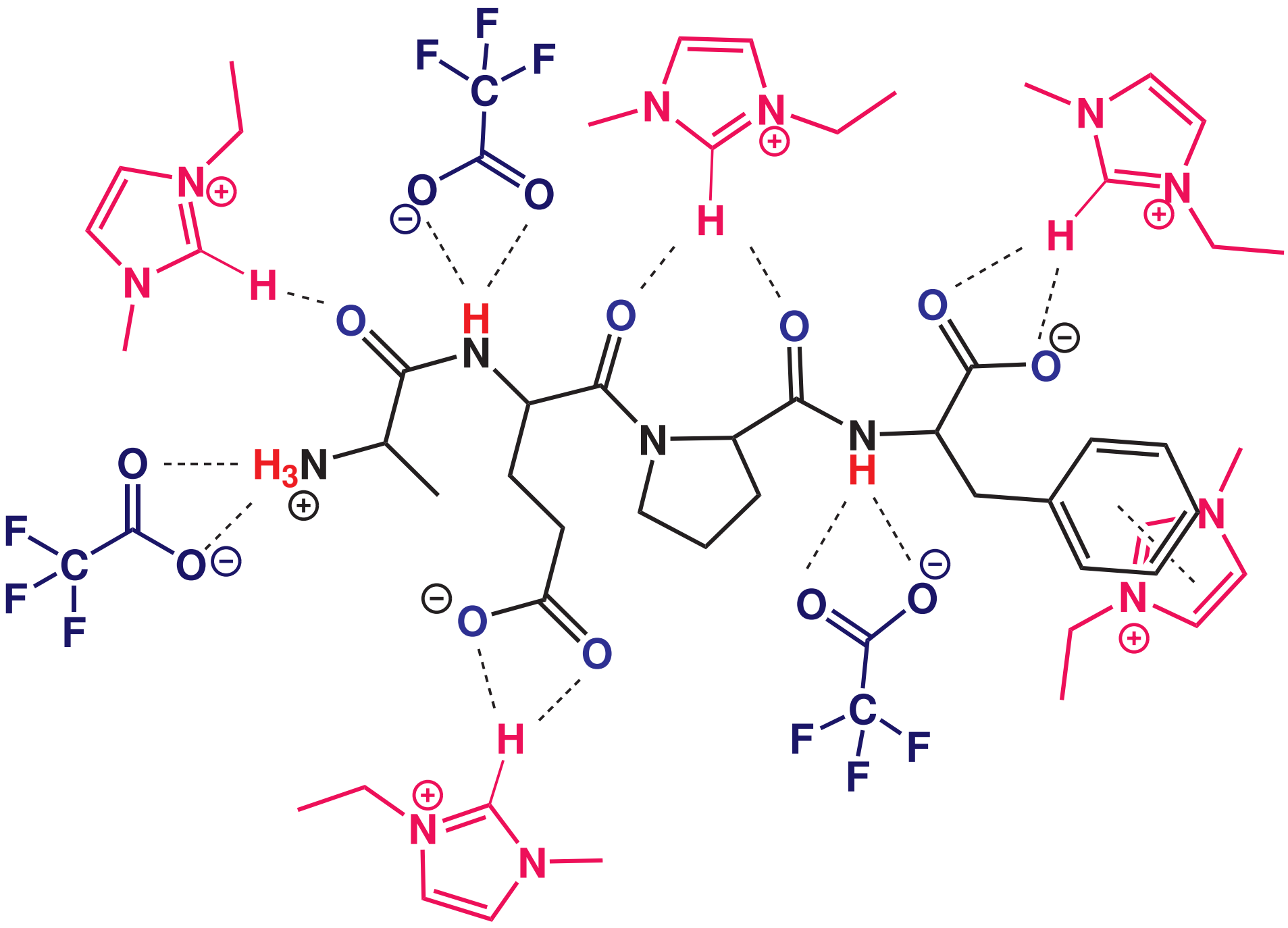


Figure S31. A representative illustration of specific hydrogen bonding and $\pi$-$\pi$ stacking interactions between an Ala-Glu-Pro-Phe peptide and constituent ions of the [EMim][TFA] ionic liquid. Refer to the main text of the article for a detailed discussion of intermolecular interactions between the peptide and the solvent molecules.
